# Influence of Waste Material Additives on the Performance of a Novel Hybrid Sol-Gel Coating on Mild Steel in 3.5% NaCl Medium

**DOI:** 10.3390/polym15132842

**Published:** 2023-06-27

**Authors:** Rami K. Suleiman, Akeem Y. Adesina, Ogunlakin Nasirudeen Olalekan, Arumugam Madhan Kumar, Fadi A. Al-Badour, Sowrirajan Subbaiah

**Affiliations:** 1 Interdisciplinary Research Center for Advanced Materials, King Fahd University of Petroleum & Minerals (KFUPM), Dhahran 31261, Saudi Arabia; adesina@kfupm.edu.sa (A.Y.A.); nasirudeen.ogunlakin@kfupm.edu.sa (O.N.O.); madhankumar@kfupm.edu.sa (A.M.K.); fbadour@kfupm.edu.sa (F.A.A.-B.); 2 Mechanical Engineering Department, King Fahd University of Petroleum and Minerals (KFUPM), Dhahran 31261, Saudi Arabia; 3 Chemistry Department, King Fahd University of Petroleum and Minerals (KFUPM), Dhahran 31261, Saudi Arabia; sowrirajan@kfupm.edu.sa

**Keywords:** coatings, corrosion, sol-gel, steel, waste

## Abstract

This article presents the synthesis of a novel hybrid sol-gel coating and its functionalization with various waste material additives. The unmodified and modified hybrid coatings were deposited on mild steel (MS) substrates, and their anticorrosion performance in a 3.5 wt.% sodium chloride corrosive environment was assessed using potentiodynamic polarization and impedance electrochemical techniques. The Fourier Transformed Infrared Spectrometry (FTIR) spectral, thermal, surface-roughness, scratch-resistance, and contact-angle characterizations were also conducted on the fabricated coatings. Electrochemical techniques proved that the coating sample loaded with the limestone additive showed the best anticorrosion behavior in the saline environment after 4 weeks of exposure. Moreover, the obtained morphological analysis data indicated better surface integrity and cross-link density for this sample compared to other waste-modified coatings. Conversely, the tire rubber and activated carbon additives showed a severe negative impact on the thermal, mechanical, and barrier properties of the parent coating, which can be attributed to the high porosity and less integrated natures of these modified coating formulations proved by their morphological images. Still, all loaded waste additives to the hybrid coating have enhanced its adhesion to the steel surface, as indicated by scratch resistance testing. Overall, the results of the present study show the need for maintaining a balance between the economic value of the modification methodology of hybrid coatings and the type of the loaded waste material additive.

## 1. Introduction

Various sectors of human life have been greatly affected by the damage of metallic infrastructures resulting from corrosion and other deterioration problems. MS is the key component of the infrastructure of various industries due to its high tensile and impact strengths, good ductility and weldability, and excellent workability with cold-forming processes [1]. However, this metallic substrate is very prone to corrosion, especially in an aggressive marine environment [2]. The dissolution of this metal in such harsh environments can be caused by the solid corrosion products accumulated on its surface [3]. The mitigation of the corrosion process on the steel surfaces has been achieved conventionally by the application of chemical phosphate and chromate conversion coatings. However, several alternatives to these coatings were proposed in the literature due to their, especially the chromium(VI)-based one, reported carcinogenic toxicity [4].

The corrosion protection of MS by hybrid sol-gel coatings has been recently considered a topic of interest for coatings scientists. Hybrid sol-gel coatings are organic-inorganic hybrid (OIH) materials that are produced by the sol-gel synthesis process, which allows the combination of inorganic and organic components in a single phase [5,6]. These coatings can provide a barrier effect that prevents contact between the metal substrate and the corrosive environment, as well as active protection by incorporating corrosion inhibitors or sacrificial anodes [6,7]. The synthesis of these hybrid materials involves the conversion of monomers (usually organometallic alkoxides) into a colloidal solution (sol) that acts as the precursor for an integrated network (or gel) of either discrete particles or network polymers. The sol undergoes hydrolysis and polycondensation reactions, which result in the formation of a gel-like network containing both a liquid phase and a solid phase. The gel then undergoes drying, which removes the solvent and causes shrinkage and densification of the network [8]. The sol-gel process allows the fine control of the coating’s chemical composition, morphology, and porosity, as well as the incorporation of dopants or functional groups [9,10]. Some of the advantages of hybrid sol-gel coatings are their low toxicity, environmental friendliness, good adhesion, low porosity, and high mechanical properties [5,6,7]. However, a need for optimizing the processing conditions as well as the coating parameters is highly crucial during the sol-gel fabrication process to avoid the production of hybrid coatings of an undesired high porous structure or less integrity on the metal surface [11].

The functionalization of hybrid sol-gel coatings with additives is a strategy to enhance the performance and functionality of the coatings for various applications. Additives can be incorporated into the hybrid sol-gel network to provide corrosion inhibition, antimicrobial activity, superhydrophobicity, or other desired features. Some examples of additives that can be used in hybrid sol-gel coatings are inhibitive pigments [12], nanoparticles [13], bacterial strains [14], organic molecules [15] and inorganic compounds [16]. The functionalization of hybrid sol-gel coatings with additives requires careful selection and optimization of the type, amount, and distribution of the additives, as well as the synthesis and processing conditions of the hybrid sol-gel system [17]. In particular, the mixing of hybrid sol-gel coatings with waste material additives is a promising way to utilize waste materials and reduce the environmental impact of the coatings. These additives can be incorporated into the hybrid sol-gel network to modify the structure, morphology, or properties of the coatings [12].

For example, the loading of a composite epoxy coating with 1–20 wt.% micronized waste tire rubber has been previously reported in the literature. The study demonstrated a positive impact of the modification step with the waste material on the mechanical and tribological properties of the epoxy base coating on carbon steel substrates [18]. The addition of nanoclay additive to a poly (amide-imide) base coating proved also to be a successful approach to enhance its adhesion strength and corrosion protection properties [19]. In another study, the corrosion and wear properties of anodic films on the surface of AA1050 aluminum alloy were found to be affected by the embedment of an eggshell bio-additive within the coating matrix [20]. Our group reported a negative impact of modifying a base hybrid sol-gel coating with some waste material additives on its anticorrosion properties on AA3003 metallic substrates exposed to a saline medium [21]. However, the effects of waste material additives on the performance and functionality of the hybrid sol-gel coatings are not well understood and may depend on the type, amount, and distribution of the additives, as well as the synthesis and processing conditions of the hybrid sol-gel system. Moreover, the amount of literature on studying the synergy between waste additives and the polymer matrix of hybrid sol-gel coatings on steel surfaces is so scarce. This makes such an area of research very challenging and open for development.

This study investigated the effect of an individual loading of a neat hybrid sol-gel polymer with five waste material additives derived from various sources (egg shells, activated carbon, waste rubber tires, limestone, and cement kiln dust) on its anticorrosion performance for MS panels exposed to a 3.5 wt.% NaCl for two weeks. In addition to this, the morphological, mechanical, and thermal analyses on the developed coatings were also examined in order to assess the compatibility between the loaded additive and the polymeric coating matrix.

## 2. Experimental Section

### 2.1. Materials

Aminopropyltriethoxysilane (APTES), Tetraethyl orthosilicate (TEOS), Dimethoxy-methyl-octadecylsilane (DMMOS), Zirconium (IV) propoxide (70% in 1-propanol), and ethanol absolute (EtOH) were obtained from Sigma-Aldrich, (St. Louis, MO, USA). Vinyltrimethoxysilane (VTMS) was obtained from Gelest Company (Morrisville, PA, USA). All chemicals utilized in this study were of analytical grade and used without further purification. The tested waste material additives were procured from various local sources.

### 2.2. Synthesis of the Parent Hybrid Coating

The parent hybrid polymeric sol-gel material (named after “C”) was prepared by mixing 10 mL of each of the precursors TEOS (9.33 g, 0.045 mol), APTES (9.46 g, 0.043 mol), DMMOS (8.60 g, 0.024 mol), VTMS (9.68 g, 0.065 mol), and 3 mL (2.19 g, 0.0067 mol) of the Zirconium (IV) propoxide precursor. The formation of the various inorganic polymeric networks in the hybrid polymer (via the hydrolysis/polycondensation reactions) was initiated by the dropwise addition of 1 mL of a 2:1 mixture of absolute ethanol-0.05 N HNO_3_ under continuous stirring. The resulting homogeneous polymeric solution was aged for 1 day in a closed beaker and under constant stirring at room temperature (RT) before modifying it with waste material additives.

### 2.3. Preparation of the Waste Additives-Loaded Hybrid Coatings

The parent hybrid coating C was further individually functionalized using five different waste-material additives (average particle size: 100 μm). This was achieved by loading 10 mL of the parent coating (C) with an optimized amount of each of the micronized waste materials listed in Table 1. The waste-modified solutions were sonicated using an ultrasonic probe (Vibracell, Sonics, Shawnee, OK, USA). The amount of waste additive, the mixing time between the additive and the parent coating, and the curing method were carefully optimized to avoid any gelification of the coating solution before it was applied to the metal surface.

### 2.4. Surface Preparation of the MS Substrates

The commercially available MS Q-panels (part no. S-36, Q-Lab Company, Westlake, OH, USA, 3 × 6 × 0.032 inches) with the chemical composition (in wt.%) (Mn (0.60% max), C (0.15% max), P (0.030% max), S (0.035% max), and Fe (remaining)) was used as a metallic substrate. The surface of the steel panels was degreased with absolute ethanol and finally dried under RT prior to the deposition of the coating formulations.

### 2.5. Deposition of Functional Coatings

**Figure 1:**
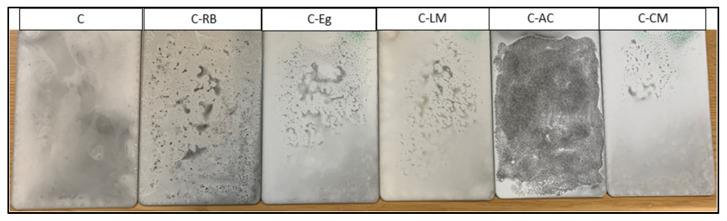
The physical appearance of the MS-coated matrices after curing.

The parent and waste-modified coatings matrices were applied to the dried steel panels using the brush technique, and the resultant uniform coatings on steel were cured using the optimum curing methodology and time listed in Table 1. An approximate and controlled thickness of 50 μm of the dry coating (measured using a PosiTector 200 coating thickness cage, Buford, GA, USA) was formed on the panels’ surface. No sign of coating delamination, pores, or cracks was visually observed on the surfaces of the cured coating layers of all formulations on the steel surface. However, the modification step with the waste additives resulted in various degrees of inhomogeneity behavior on the surface of the cured samples, especially for the rubber and activated carbon additives (Figure 1). The various clusters formed on the surface of coated samples can be attributed to the presence of Si-O-Si networks of different sizes.

### 2.6. Characterization of Coatings

The structural characterization of the C-coating formulations was analyzed by a Thermo Scientific Nicolet IS5 Fourier Transformed Infrared Spectrometer using the Attenuated total reflection (ATR) mode and in the observation range of 600–3600 cm^−1^. The thermal degradation behavior of the cured coating layers on MS substrates was investigated using the TA SDT650 simultaneous thermal analyzer (TA Instruments, New Castle, DE, USA) under a nitrogen flow of 200 mL min^−1^ and a heating rate of 20 °C min^−1^ up to 690 °C.

The surfaces of the deposited coatings on MS were morphologically characterized using a scanning electron microscope (SEM) JEOL JSM-6610 LV coupled with an energy-dispersive X-ray spectrometer (EDS). Static water contact angle (WCA) measurements were performed at room temperature using an optical contact angle meter (Biolin Scientific, Theta lite, Espoo, Finland). A 5 μL drop of distilled water was deposited on different positions of the surface of cured coated samples, three measurements were performed on each sample, and the mean values were recorded. An optical profilometer (Profilm 3D, Filmetrics, San Diego, CA, USA) was used to measure the microscale roughness of the surfaces. Three images with a pixel resolution of 1632 × 786 were obtained from different locations on the samples to calculate the surface roughness values. The quantification of “surface roughness” can be performed using a selection of different parameters, such as the root mean square height of the surface (Rq), the maximum height of peaks (Rp), maximum depth of valleys (Rv), Maximum peak to value height (Rpv), and arithmetic average height of the surface (Ra).

The anti-corrosive performance assessment of the C-coating formulations on MS panels after 24 h and 4 weeks of exposure to 3.5 wt.% NaCl solution was achieved using electrochemical impedance spectroscopy (EIS) and DC-polarization measurements (Gamry Reference 620 potentiostat/galvanostat, USA). The experimental EIS data of the coated samples were simulated using the Echem Analyst software (version 6.04). The EIS measurements were conducted at the open circuit potential (OCP), a frequency range of 100 kHz–10 mHz, and a perturbation voltage of 10 mV. These experiments were conducted in a typical three-electrode cell with the coated steel sample as a working electrode, a graphite rod as the counter electrode, and the saturated calomel electrode (SCE) as the reference electrode. The masked area of the sample that was exposed to the saline solution was 10 cm^2^. The potentiodynamic polarization curves were measured at a potential range of ±0.25 V, a scan rate of 0.5 mV/s, and a sample period of 1 s. Corrosion current (i_corr_) and corrosion potential (E_corr_) were calculated using the Gamry Framework and Echem Analyst software (version 6.04) of the potentiostat.

The nanoindentation hardness was performed on a STEP 500 Instrument Indentation Measurement System from Anton Paar Instruments, Switzerland. A Berkovich diamond indenter was used with a normal load of 50 mN for 10 s dwelling time. The equipment accuracy was very high, and the measurement was repeated nine times on each sample. The average hardness values are presented. The scratch test was conducted using the standard Rockwell diamond indenter with a 100 μm tip radius. The indenter was pressed against the coating with an initial applied load of 30 mN and then pulled across the coating surface with progressive loading until the maximum applied load of 10 N was attained. The scratch test parameters utilized over a scanning length of 5 mm were a 2.5 N min^−1^ loading rate and a 1.25 mm min^−1^ scratch traverse speed, respectively. During the test, the normal load, penetration depth, acoustic emission (AE), frictional force, and coefficient of friction (COF) were measured.

## 3. Results and Discussion

The precursors involved in the synthesis of the parent hybrid polymer “C” were selected inspired by their individual involvement in various previously reported hybrid sol-gel polymeric coatings in the literature [22,23,24]. Our hybrid material was fabricated at room temperature and pressure via hydrolysis and polycondensation of organically functionalized silane and zirconium(IV) propoxide precursors. The APTES precursor helped the hybrid polymer to crosslink further at room temperature, while Zr induced more toughness in the hybrid polymer [25]. Adding ethanol was also planned to enhance the miscibility of the precursors and water, which induces more homogeneity in the final hybrid polymeric solution [26].

Motivated by the excellent literature on mixing hybrid sol-gel coatings with useful additives, [10,12,15] we loaded the hybrid polymer “C” with various waste material additives, deposited them on MS panels, and finally cured them using an optimized curing method (see Section 2.5). The coatings were then subjected to structural, thermal, morphological, corrosion protection, and scratch resistance tests.

### 3.1. FTIR Analysis of the Neat and Modified Hybrid Polymers

**Figure 2:**
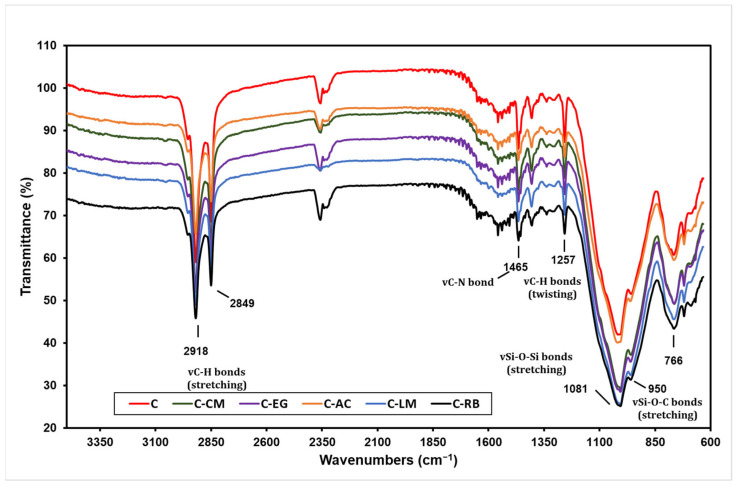
FTIR analysis of the neat and waste material-modified hybrid sol-gel polymers.

The structural characterization of the base and waste material-modified hybrid polymers was attained by The FTIR analysis. This technique can provide information about the chemical bonds, molecular structure, and composition of the hybrid sol-gel network. It can also reveal the presence and amount of organic and inorganic components, as well as their interactions and transformations during the sol-gel process and subsequent treatments [27]. The FTIR spectra of all coating matrices prepared in this study are presented in Figure 2 (The individual spectra of all samples are available in Section S1 in the Supplementary Materials). The formation of the Si-O-Si inorganic network in the polymer network can be deduced from the strong peak at 1081 cm^−1^ in all spectra [28]. The presence of a small adjacent peak at 950 cm^−1^ can correspond to the Si-O-C stretching vibrations, which can indicate the formation of covalent bonds between the organic and inorganic phases or the presence of different types of siloxane units, such as Qn (where n is the number of bridging oxygens), Tn (where n is the number of non-bridging oxygens), or Dn (where n is the number of geminal oxygens) in the hybrid polymer [29]. The two peaks at 2849 and 2918 cm^−1^ in the spectra of “C” are attributed to the C-H stretching vibrations, which can indicate the presence of different types of organic groups in the hybrid sol-gel network of this sample [21]. The peak at 1257 cm^−1^ corresponds to the twisting vibration of the C-H bond in the hybrid polymer. The absence of any broad peak in the 3200–3600 cm^−1^ region in the spectra that can be ascribed to the O-H stretching vibrations reveals the absence of any amount of water, free hydroxyl groups, or hydrogen bonds in the cured hybrid sol-gel network. This indicates a complete degree of hydrolysis, condensation, or dehydration of our developed sol-gel systems [30]. It is worth mentioning here that the modification step of the parent hybrid polymer with waste additives did not induce any significant change in its FTIR spectra, which reveal a composite nature for the modified polymers [31].

### 3.2. Thermal Analysis of the Coatings Matrices

**Figure 3:**
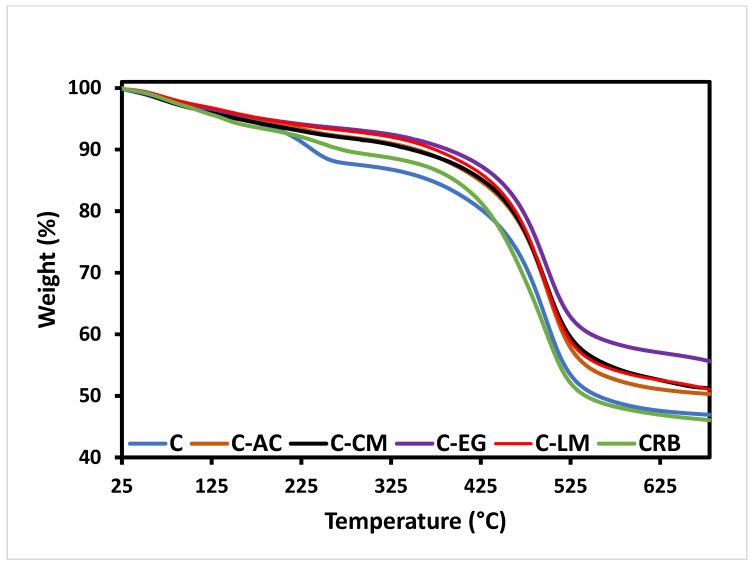
TGA plots of the S-coating matrices.

The thermal properties of the cured neat polymer, as well as the waste-modified composite materials, were analyzed by TGA. This technique can provide valuable information on the thermal stability, decomposition, and weight loss of the hybrid sol-gel network. It is very clear from the TGA profiles of all coating matrices depicted in Figure 3 that all the waste additives (except for the tire rubber) have remarkably increased the thermal stability of the parent hybrid polymer. The samples modified with cement and limestone additives showed very comparable weight loss behaviors (Figure 3). The C-EG sample demonstrated the least degradation behavior compared to the other samples indicating excellent reinforcing and crosslinking properties of this additive with the bulk matrix of the parent hybrid polymer. The parent polymeric material (sample C) exhibited more degradation behavior between 200–300 °C compared with the modified polymer, which reveals the occurrence of either ethanol/water evaporation or further condensation in the inorganic polymeric network [32].

### 3.3. Morphological Analyses of the Coating Formulations on Steel

**Figure 4:**
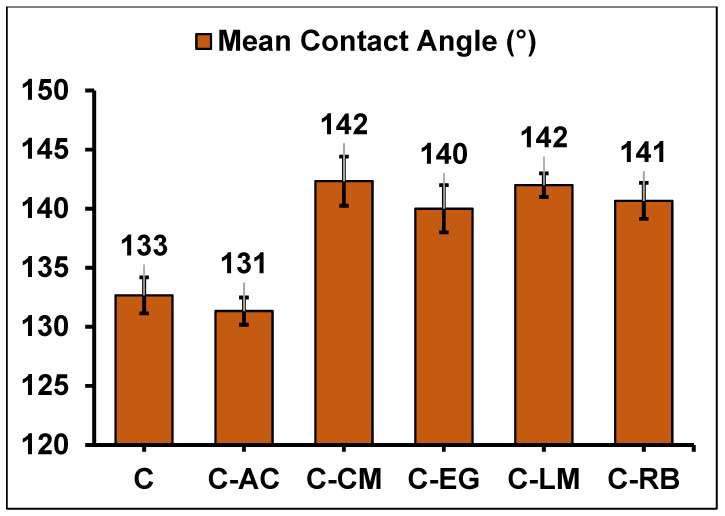
Water contact angle (CA) values (in °) of the cured C-coating matrices on MS substrate.

Morphological characteristics of hybrid sol-gel coatings on steel are the features that describe the shape, size, and structure of the hybrid sol-gel coatings on the steel surface. Morphological characteristics can affect the optical, mechanical, chemical, and functional properties of the hybrid sol-gel coatings, such as transparency, adhesion, corrosion resistance, or hydrophobicity [12]. In order to get insight into the hydrophilic/hydrophobic properties of the cured parent hybrid coating “C” and the waste-modified ones, we have analyzed the coating films on steel using the water contact angle (WCA) measurements and the obtained values are depicted in Figure 4 (Representative WCA images of all coated samples are available in Section S2 in the Supplementary Materials). Contact angle analysis is a technique used to measure the wettability of a surface, which can ultimately affect its readiness for corrosion onset. The wettability properties of a coating layer on a metal surface are highly dependent on the chemistry of its precursor as well as the doped additives [33]. The data in Figure 4 revealed excellent hydrophobic properties for all coating matrices prepared in this work (WCA > 130°), which can be attributed to the DMMOS silane precursor that has a long C18 alkyl chain in its chemical structure and characterized by low surface energy properties [24]. Moreover, the loading of patent hybrid polymer matrix with waste additives yielded a minor enhancement (WCA ~140°) in the hydrophobic properties of these coatings. This might be explained by the change in the surface roughness properties to be described below.

**Figure 5:**
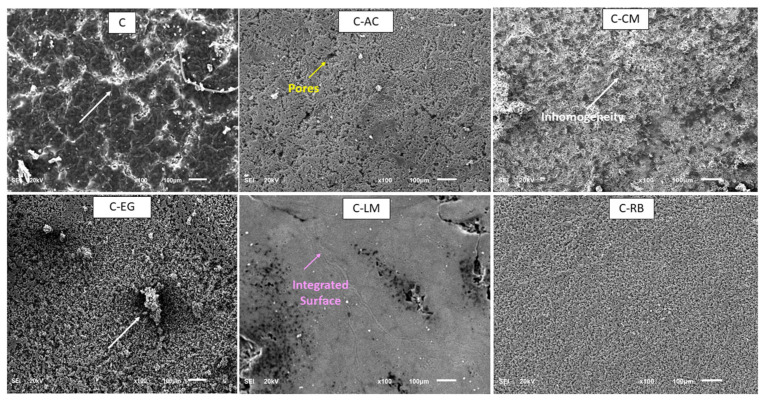
SEM micrographs of C-coated samples on MS.

The shape and integrity of the developed cured coated matrices on steel panels were analyzed by the SEM technique, and the obtained top-surface images are presented in Figure 5. The images depicted a high degree of inhomogeneity on the surface of samples C, C-EG, and C-CM, while some pores can be seen on the surface of samples C-AC and C-RB. This indicates low compatibility and homogeneity for these two additives with the bulk matrix of the hybrid polymer. The surface of the coating layer modified with the limestone additive (C-LM) was the only one that showed an appreciated level of continuity and integrity on the steel surface, which suggests more compatibility for this additive with the parent hybrid polymer compared with other additives. Energy-dispersive X-ray spectra of all samples were also collected and presented as Section S3 in the Supplementary Materials. The spectra confirmed the presence of the elements of the hybrid polymer.

Next, the surface roughness analysis of the parent and waste-modified coatings on steel was achieved by the optical profilometry test. This technique can provide information about the surface topography and texture of the hybrid sol-gel coatings. Optical profilometry can measure the surface height variations and calculate the surface roughness parameters, such as the root mean square height of the surface (Rq), the maximum height of peaks (Rp), the maximum depth of valleys (Rv), the maximum peak to value height (Rpv), and the arithmetic average height of the surface (Ra). In general, Ra (average surface roughness) and Rq (root mean square; RMS) are the important and frequently considered factors for analyzing surface roughness [18]. The obtained roughness parameters for all samples are listed in Table 2, whereas a typical surface roughness image for each coating sample is also available in the Supplementary Materials (Section S4). It can be observed from a comparison of the surface roughness between the base “C” and waste-containing coating matrices on steel (Table 2) that the modification step resulted in a moderate increase in the roughness of all samples except for sample C-AC which showed a drastic change in its surface roughness behavior resulted from the low homogeneity behavior of this sample with the parent coating. The roughness values for all samples are in the micrometer scale, which suggests a moderate co-solubility behavior for the waste additives (except the activated carbon) with the polymeric matrix of the hybrid base coating. The lowest Rpv value for sample C-LM indicates a high degree of film smoothness for this coating on the steel surface. It is worth mentioning here that the increase in the surface roughness of the waste-functionalized steel-coated samples might also contribute to the enhancement in the hydrophobic properties of these samples [34].

### 3.4. Mechanical Testing

The mechanical strength, wear resistance, and durability of the hybrid sol-gel coatings can be assessed by the indentation hardness measurements. This analysis can provide valuable information on the resistance of the hybrid sol-gel coatings to permanent deformation or penetration by an indenter. The hardness properties of hybrid sol-gel coatings are versatile to various factors such as the chemistry of precursors, preparation conditions, and the modification of the hybrid sol-gel coating systems with functional additives [35]. The Elastic indentation modulus (E_IT_) and the indentation hardness (H_IT_, at 50 mN) of parent and waste-modified coatings on steel are summarized in Table 3. It can be noticed from the results that the modification of the parent hybrid sol-gel coating with the activated carbon and tire rubber resulted in a noticeable reduction in its hardness properties, which might be caused by imparting more organic content into the hybrid polymer by the two additives. In contrast to this behavior, the other additives showed an advantageous role in enhancing the hardness properties of the parent coating as a result of their higher inorganic material content. The highest hardness properties were found for the C-LM sample, which can be added to the other desired properties for this coating formulation reported above. On the other hand, the low hardness properties for the C-RB sample indicate a significant negative impact of this additive on the mechanical strength, densification, or dispersion of this hybrid sol-gel network.

**Figure 6:**
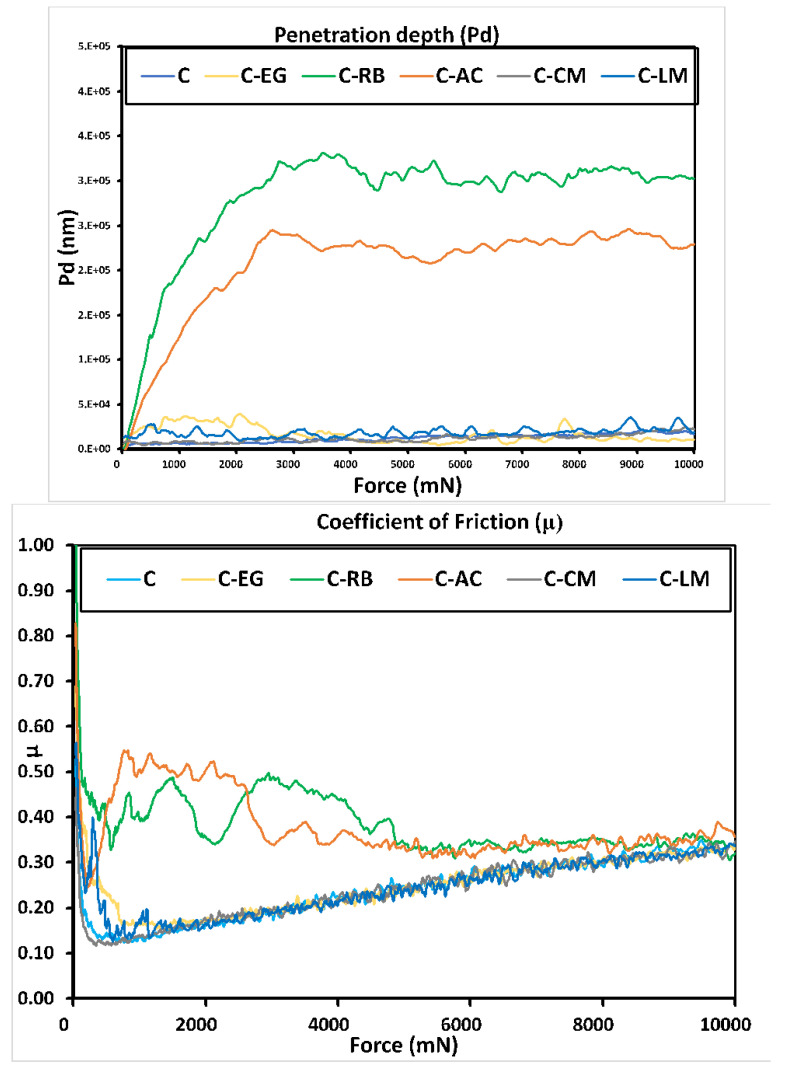
Penetration depth (**Top**) and coefficient of friction (**down**) plots of unmodified and modified coating matrices on MS surfaces.

The scratch resistance properties of all the coating matrices on steel were also examined in order to evaluate and compare the degree of adhesion and the presence of any coating’s delamination or cracking behaviors in the coated films. The penetration depth and coefficient of friction plots of all coating matrices on MS surfaces are illustrated in Figure 6, and the obtained critical load (*L_c_*) values are recorded in Table S1 in the Supplementary Materials. Any increase in the penetration depth is usually associated with an increase in the critical load. This is evident with the waste-loaded coatings that showed higher critical load values in comparison with the parent coating, which reveals an enhancement in the degree of bonding of these coating formulations to the steel surface [24]. This result reflects an advantageous aspect of modifying our developed hybrid sol-gel coatings with the waste additive. As the penetration is increased, a larger area of the coating material is exposed to the scratching force, making it more susceptible to damage. Samples C-CM, C-EG, and C-RB demonstrated comparable and relatively low *L_c_* values indicating less compatibility between these additives and the matrix of the hybrid polymer.

### 3.5. Electrochemical Assessment of the Anticorrosion Performance of the Hybrid Coating Systems on Steel Substrate

**Figure 7:**
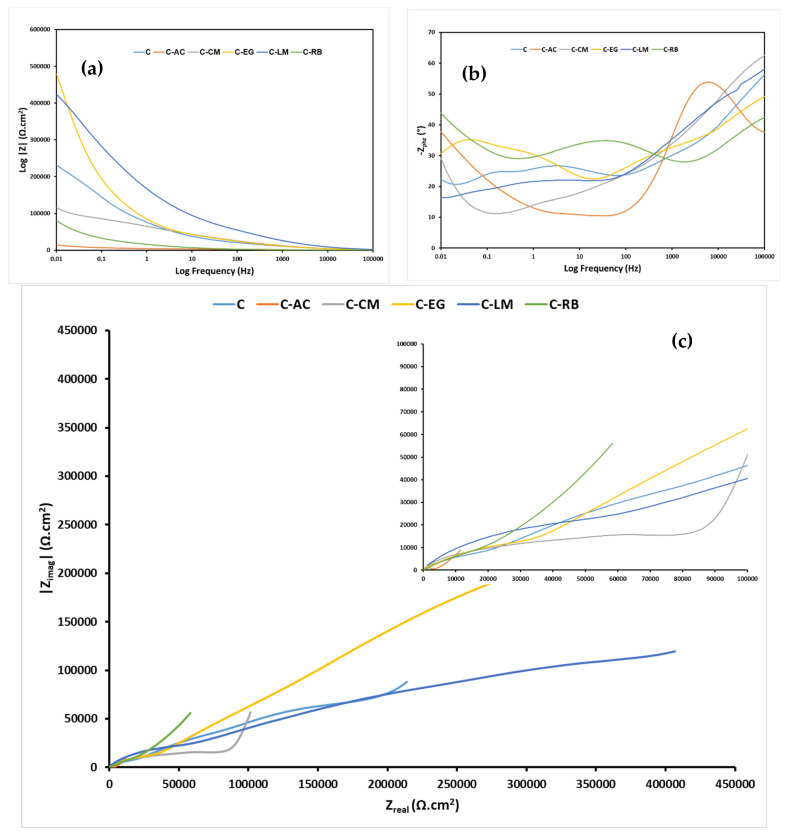
EIS (**a**) Bode-resistance, (**b**) Bode-phase, and (**c**) Nyquist plots of all coating matrices on MS substrates after *24* h of exposure to the 3.5 wt.% NaCl solution.

**Figure 8:**
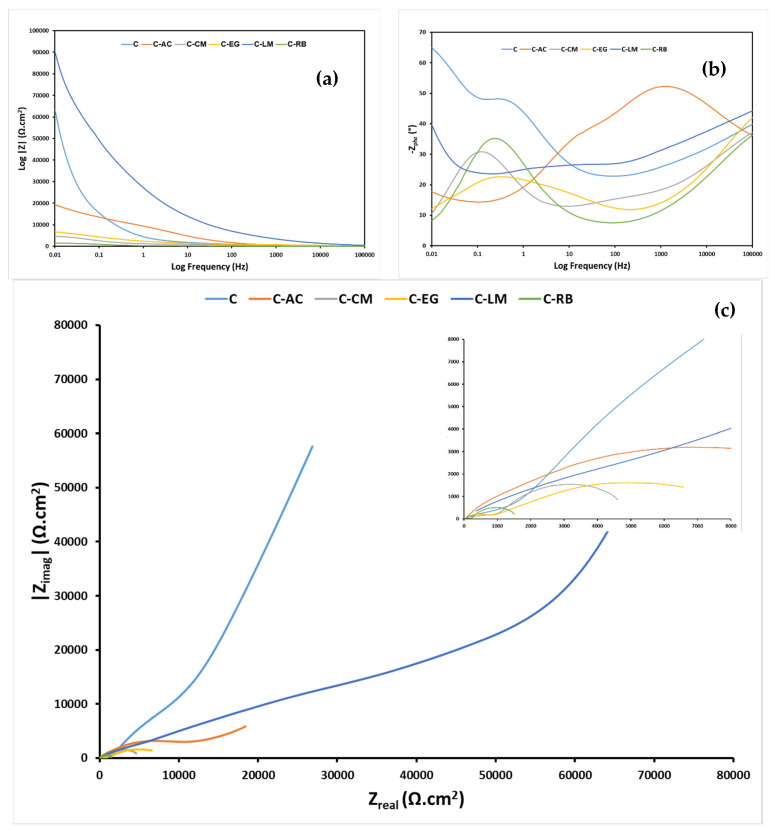
EIS (**a**) Bode-resistance, (**b**) Bode-phase, and (**c**) Nyquist plots of all coating matrices on MS substrates after *4 weeks* of exposure to the 3.5 wt.% NaCl solution.

The corrosion protection performances of the parent and waste-modified coating matrices on steel panels were assessed by exposing them continuously to 3.5 wt.% NaCl corrosive medium followed by electrochemical and visual observation tests. The bode, bode-phase, and Nyquist electrochemical impedance spectroscopy (EIS) of the steel-coated samples after 24 h and 4 weeks of exposure to the saline medium are presented in Figure 7 and Figure 8, respectively. The individual Nyquist spectra of all samples at the two immersion times are also available as Sections S5 and S6 in the Supplementary Materials. EIS is a powerful technique used to characterize the electrical properties of materials and, more commonly, in studying the corrosion of metals and coatings [36,37,38].

At early times of immersion in the saline medium, the samples loaded with the limestone (C-LM) and eggshell (C-EG) showed higher impedance values than those for the parent hybrid coating (Figure 7a,b), which indicates enhanced barrier properties as a result of the modification step with the aforementioned two waste additives. This enhancement can also be proved by the presence of wider semicircles in the EIS Nyquist plots of the two waste-modified samples compared with the unmodified one (Figure 7c). In order to achieve a clearer visualization of the EIS behavior of the coated samples, the high-frequency region of the Nyquist curves was further enlarged. An increase in the impedance values in the EIS Bode and the width of the semicircles in the Nyquist plots are usually revealing an enhanced corrosion protection performance for hybrid sol-gel coatings. Still, an acceptable corrosion protection performance can be expected for the other waste-functionalized samples considering their high obtained impedance values (about 10^5^ Ω.cm^2^) after 24 h of immersion (Figure 7).

The impact of the waste additives on the corrosion-resistance performance of the parent hybrid sol-gel coating at prolonger immersion times can be deduced from the EIS data plotted in Figure 8. The Bode-phase (Figure 8b) and Nyquist (Figure 8c) plots of the EIS data of all steel-coated samples clearly showed the presence of three time constants. The one in the high-frequency range corresponds to the behavior of the hybrid film, the one in the middle-frequency range corresponds to the behavior of the oxide layer on the surface, and the last one at low frequencies corresponds to the behavior of interphase with the metal surface [39]. The EIS plots demonstrate clearly that only sample C-LM has affected in a positive manner the barrier properties of the neat parent hybrid coating. The lower impedance values of the remaining modified samples compared with the unmodified coating suggest a deterioration in the barrier properties of these coating systems on the steel surface after 4 weeks of immersion in the saline medium. It can be seen from the plots that the corrosion protection performance of the parent hybrid polymer was found to give the worst behavior after its functionalization with the tire rubber additive suggesting the lack of any synergy between the two components.

**Figure 9:**
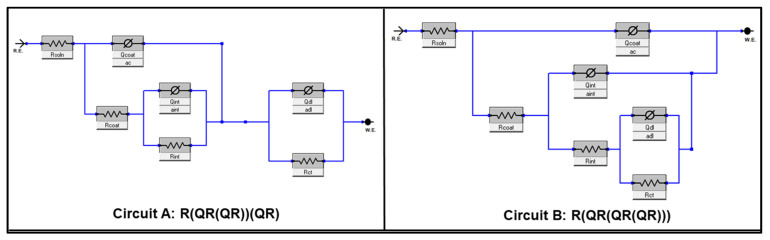
Electrochemical equivalent circuits used to fit EIS data of the coating matrices on the MS substrate.

The experimentally collected EIS data of all steel-coated samples were further modeled using the equivalent circuit (EC) models shown in Figure 9, and the obtained fitting results are also listed in Table 4. The proposed ECs involve a solution resistance (R_s_), a time constant of coating containing a resistance (R_coat_) and a constant phase element (Q_coat_) at high frequencies, a [Q_int_R_int_] component corresponding to an intermediate coating layer, and a time constant attributed to the charge transfer resistance of the metal (R_ct_) and a double-layer constant phase element (Q_dl_) at low frequencies. The quality of fitting was judged based on the obtained chi-square (χ^2^) value for each EC; a value of 10^−3^ for χ^2^ and lower usually refers to excellent fitting [40]. Circuit A was found to be suitable for fitting the EIS data of all samples at the two immersion times, except for samples C-EG and C-LM, in which Circuit B was found to give the best fit for the two sets of data after 4 weeks of immersion. In the circuits, the capacitor component was replaced with a constant-phase element (CPE, Ø) was considered to account for the non-perfect capacitive behavior of the coating as a result of double layers inhomogeneity or non-uniformity in coating thickness [16]. The Qdl and Rct elements are usually used to rank the corrosion resistance performance of coated samples on metal surfaces. The relatively high resistance and low capacitance values of sample C-LM after 4 weeks of immersion (Table 3) reveal a stronger corrosion protection performance for this coating formulation in comparison with the other developed coating formulations in this work. Moreover, the low R_coat_ values of all waste-embedded samples (the highest value is for C-LM) suggest the presence of high porosity, water absorption, and coating degradation in these samples. Still, sample C-LM suffered from a reduction in its R_coat_ value, which is expected to occur as a function of immersion time. Any increase in this parameter may result from the blockage of defects or pores by corrosion products. Overall, the rank of the corrosion protection performance of the developed coating systems is C-LM > C > C-AC > C-EG > C-CM > C-RB.

**Figure 10:**
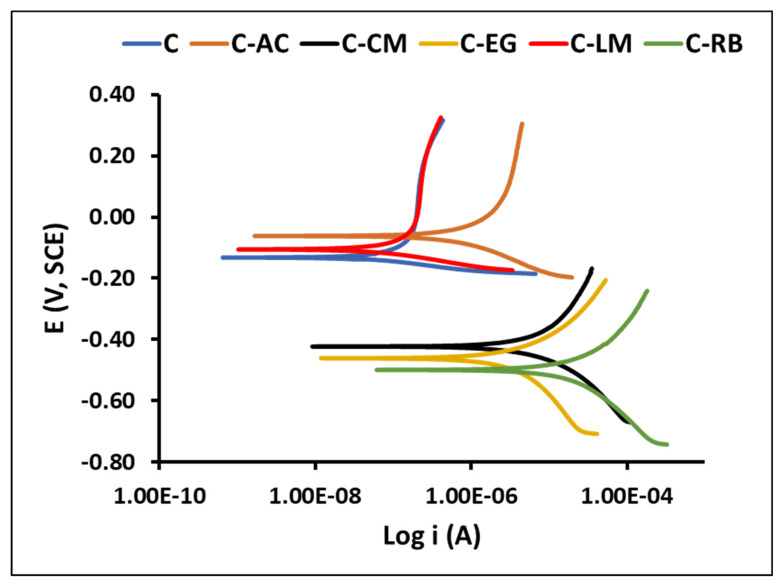
Potentiodynamic polarization curves of the C matrices-coated specimens after *4 weeks* of immersion in 3.5 wt.% NaCl solution.

We have also conducted the DC-potentiodynamic polarization analysis on the coated matrices on steel after 4 weeks of immersion in the saline solution targeting to give further support to the EIS analysis reported above. The obtained polarizing curves are shown in Figure 10, and the measured electrochemical corrosion parameters (using the Tafel extrapolation treatment, corrosion potential, E_corr_, corrosion current density, and i_corr_) and corrosion rate of parent and waste-modified steel-coated coatings after 4 weeks of exposure to a 3.5 wt.% NaCl solution are available in Table S2 in the Supplementary Materials. The analysis of the data showed very low and comparable i_corr_ values for the C and C-LM samples indicating the best corrosion-resistance properties for the limestone-modified coating. Moreover, the most negative E_corr_ value of sample C-RB suggests a poor protection performance for this coating formulation. Overall, the polarization results agree perfectly with the EIS testing results.

**Figure 11:**
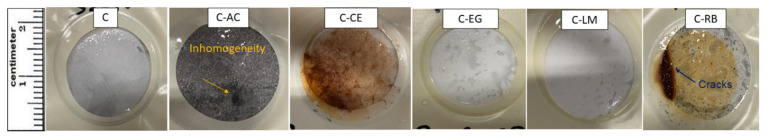
Photo-digital images of the MS-coated samples after exposure to the 3.5 wt.% NaCl for 4 weeks.

Figure 11 depicts the photo-digital images of the parent and waste-modified steel-coated matrices after four weeks of exposure to the saline medium. The images illustrate the presence of a small number of cracks/defects in the coating layers of samples C-CE and C-RB, which might explain their low barrier properties reported above. In contrast to this, the surfaces of the parent coating “C” and the waste-modified samples C-EG and C-LM were intact with no defects or delamination phenomena, indicating an appreciated corrosion-resistance behavior for the two samples. Moreover, the observed inhomogeneity in C-AC remained linked and integrated after the exposure test. Overall, the visual observation results showed an excellent agreement with the electrochemical and morphological testing results reported earlier in this paper.

## 4. Conclusions

This study demonstrated the effects of waste material additives on the performance and functionality of the hybrid sol-gel coatings for protecting MS substrates in a 3.5 wt.% NaCl medium. The mechanical, thermal, and barrier properties were found to depend greatly on the type, amount, and distribution of the additives, as well as the synthesis and processing conditions of the hybrid sol-gel system. In particular, the coating sample functionalized with the limestone additive was found to yield the best enhancement in the desired properties of the parent hybrid coatings, which can be attributed to its reinforcement role. On the other hand, the tire rubber additive showed a major deterioration in the integrity and homogeneity of the network of the parent hybrid polymer. In conclusion, our study proved that waste material additives can:

- Provide a low-cost and eco-friendly source of fillers, pigments, or modifiers for the hybrid sol-gel coatings;
- Enhance the roughness, hydrophobicity, or adhesion of the hybrid sol-gel coatings and;
- Improve the mechanical strength, electrical conductivity, or thermal stability of the hybrid sol-gel coatings by forming a percolating network or acting as a catalyst within the matrix.

On the other hand, waste material additives can:

- Negatively affect the corrosion resistance, barrier properties, or homogeneity of the hybrid sol-gel coatings by introducing defects, impurities, or incompatibilities in the network, and;
- Increase the complexity and variability of the hybrid sol-gel system, making it more difficult to optimize and control the coating parameters and properties.

The waste-modified hybrid sol-gel coating systems developed in this study can be considered a promising alternative to commercial toxic chromate and phosphate conversion coatings.

## Figures and Tables

**Table 1:** Details on the coating matrices prepared in this work.

Sample Code	Additive (Amount in g)	Sonication Time (in min.)	Curing Methodology (Time in h)
C	-	-	RT (2)
C-AC	Activated carbon (0.25)	0.5	Oven (24)
C-CM	Cement (0.2)	2.5	RT (6)
C-EG	Eggshell (0.5)	5	Oven (24)
C-LM	Limestone (0.2)	2.5	RT (6)
C-RB	Tires rubber (0.5)	5	Oven (24)

**Table 2:** Roughness parameters (Mean ± STD) of unmodified and waste material-modified coatings.

Sample	Ra (μm)	Rq (μm)	Rp (μm)	Rv (μm)	Rpv (μm)
C	1.891 ± 0.263	2.415 ± 0.247	13.655 ± 1.01	32.270 ± 3.286	45.925 ± 16.746
C-AC	5.408 ± 0.817	7.524 ± 0.111	10.801 ± 1.678	35.100 ± 6.616	45.901 ± 8.297
C-CM	2.214 ± 0.456	2.982 ± 0.702	6.467 ± 0.837	19.260 ± 5.073	25.727 ± 8.339
C-EG	2.103 ± 0.326	2.866 ± 0.356	6.876 ± 0.902	21.950 ± 2.510	28.826 ± 2.385
C-LM	2.136 ± 0.313	2.719 ± 0.357	7.724 ± 1.553	13.637 ± 1.724	21.360 ± 10.044
C-RB	2.596 ± 0.512	3.370 ± 0.621	8.221 ± 0.702	26.518 ± 6.422	34.738 ± 6.002

Ra: arithmetic average height of the surface; Rq: root mean square height of the surface; Rp: maximum height of peaks; Rv: maximum depth of valleys; and Rpv: Maximum peak to value height; STD: Standard Deviation.

**Table 3:** Elastic indentation modulus (E_IT_) and the indentation hardness (H_IT_, at 50 mN) of unmodified and waste material-modified coating matrices on the steel surface.

Samples	E_IT_ [GPa]		H_IT_ [MPa]	
	Average	STD	Average	STD
C	0.554	0.049	4.460	0.389
C-AC	0.426	0.063	13.137	2.5413
C-CM	1.351	0.101	5.1005	1.654
C-EG	1.255	0.171	4.044	1.184
C-LM	2.373	0.105	15.357	3.435
C-RB	0.202	0.030	3.351	0.691

**Table 4:** Fitted parameter values of C-coated samples using EC presented in Figure 9.

Immersion Time	Parameter	Sample					
		C	C-AC	C-CM	C-EG	C-LM	C-RB
24 h	Rsoln (Ω)	46.1	10.0	19.1	10.0	5.0	10.0
	Rcoat (kΩ)	39.91	4.83	72.58	4.05	35.36	0.67
	Qcoat (μF cm^−2^ s^−(1−αc)^)	3.27	90.04	2.92	0.045	0.075	1.39
	acoat	0.5	0.3	0.5	0.7	0.6	0.6
	Rint (MΩ)	14.93	3.77	0.19	0.024	0.9	13.9
	Qint (μF cm^−2^ s^−(1−αc)^)	7.11	257.90	170.30	1.16	3.29	77.19
	aint	0.2	0.6	0.9	0.5	0.3	0.6
	Rct (kΩ)	10.93	1.79	16.79	2.03 × 10^3^	2.16 × 10^3^	22.42
	Qdl (μF cm^−2^ s^−(1−αc)^)	0.25	0.12	0.16	6.68	139.00	18.82
	adl	0.6	0.9	0.7	0.5	0.8	0.5
	χ^2^ (×10^3^)	1.68	0.22	0.067	0.14	0.21	0.059
	Circuit	A	A	A	A	B	A
4 Weeks	Rsoln (Ω)	14.0	15.0	20.0	15.0	15.0	10.6
	Rcoat (kΩ)	1.19	0.30	0.24	0.92	4.92	0.11
	Qcoat (μF cm^−2^ s^−(1−αc)^)	20.02	2.09	1.31	2.74	1.68	2.25
	acoat	0.5	0.9	0.7	0.6	0.5	0.7
	Rint (kΩ)	10.37 × 10^3^	132.90	1.06	0.12	129.20	0.13
	Qint (μF cm^−2^ s^−(1−αc)^)	225.90	349.2	160.80	3.07	16.33	435.90
	aint	0.9	0.4	0.4	0.9	0.4	0.4
	Rct (kΩ)	20.94	11.29	3.900	8.39	2.51 × 10^3^	1.44
	Qdl (μF cm^−2^ s^−(1−αc)^)	88.48	17.43	715.00	232.10	99.11	1.26 × 10^3^
	adl	0.6	0.6	0.8	0.5	0.9	0.8
	χ^2^ (×10^3^)	1.32	0.28	0.021	0.28	0.15	0.16
	Circuit	A	A	A	B	B	A

## Data Availability

The data presented in this study are available on request from the corresponding author.

## Supplementary Materials

The following supporting information can be downloaded at: https://www.mdpi.com/article/10.3390/polym15132842/s1.
