# Supplementary material for: Influence of Waste Material Additives on the Performance of a Novel Hybrid Sol-Gel Coating on Mild Steel in 3.5% NaCl Medium
Source: Polymers (Basel). 2023 Jun 27;15(13):2842. doi: 10.3390/polym15132842 (PMC10346716; doi:10.3390/polym15132842)
Supplement: Supplementary file 1 [file polymers-15-02842-s001.zip › polymers-2436173-supplementary.pdf]

SUPPORTING INFORMATION

For

Influence of Waste Material Additives on the Performance of a Novel Hybrid Sol-Gel Coating on Mild Steel in 3.5% NaCl Medium

Rami K. Suleiman[a]*, Akeem Y. Adesina[a], Ogunlakin Nasirudeen Olalekan[a], A. Madhan Kumar[a], Fadi A. Al-Badour[a,b], Sowrirajan Sowrirajan[c]

[a] Interdisciplinary Research Center for Advanced Materials, King Fahd University of Petroleum & Minerals (KFUPM), Dhahran 31261, Saudi Arabia

[b] Mechanical Engineering Department, King Fahd University of Petroleum and Minerals (KFUPM), Dhahran 31261, Saudi Arabia

[c] Chemistry Department, King Fahd University of Petroleum and Minerals (KFUPM), Dhahran 31261, Saudi Arabia

	**Contents**	**Page #**
**S1.**	FTIR spectra of the **C** coating formulations.	**2**
**S2.**	Representative water contact angle images of all **C** mild steel-coated matrices.	**5**
**S3.**	Top-surface micrographs of all **C** mild steel-coated matrices immersed in 3.5 wt.% NaCl for 4 weeks and the EDS analysis of the coating layer of the samples.	**6**
**S4.**	Surface roughness typical optical images of all **C** coating matrices on steel using a 3D profilometer.	**8**
**S5.**	Individual Nyquist spectra of all **C** coating matrices after **24 h of** exposure to the 3.5 wt.% NaCl corrosive medium.	**11**
**S6.**	Individual Nyquist spectra of all **C** coating matrices after **4 weeks of** exposure to the 3.5 wt.% NaCl corrosive medium.	**14**
**Table S1**	Critical load (L_c) for the developed parent and waste-modified mild steel hybrid-coated samples in this study.	**17**

Table S2 Electrochemical corrosion parameters (corrosion potential, E_{corr}, corrosion current density, i_{corr}) and corrosion rate of mild steel coated with the **C** hybrid sol-gel coatings after *4 weeks* of exposure to a 3.5 wt.% NaCl solution. **18**

Figure S1. FTIR spectra of the **C** coating formulations

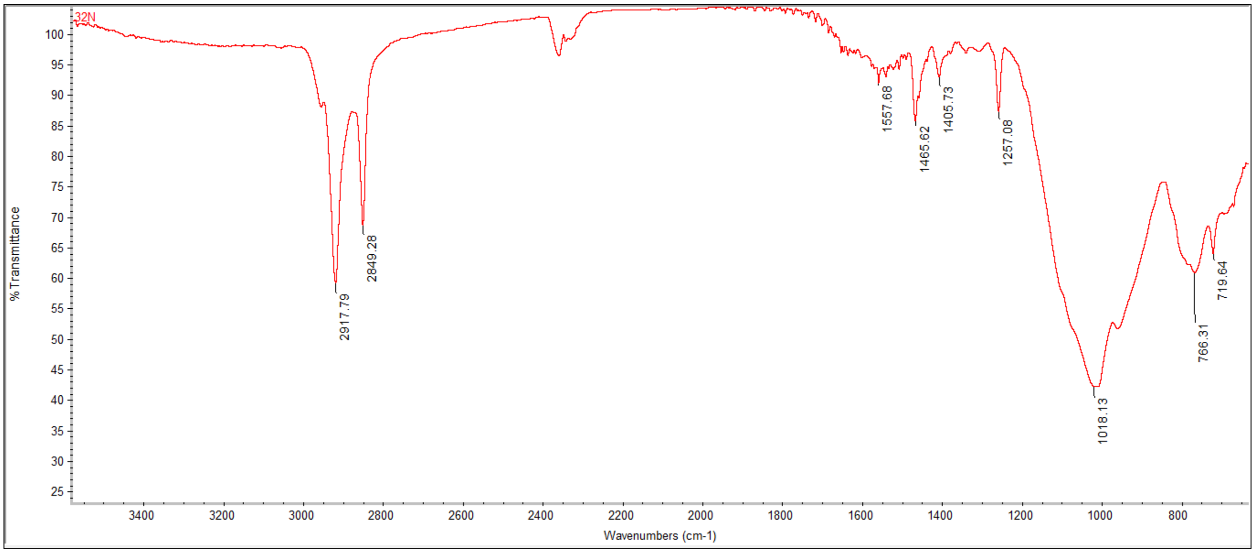

Figure S1-1. FTIR spectrum of the **C** sample.

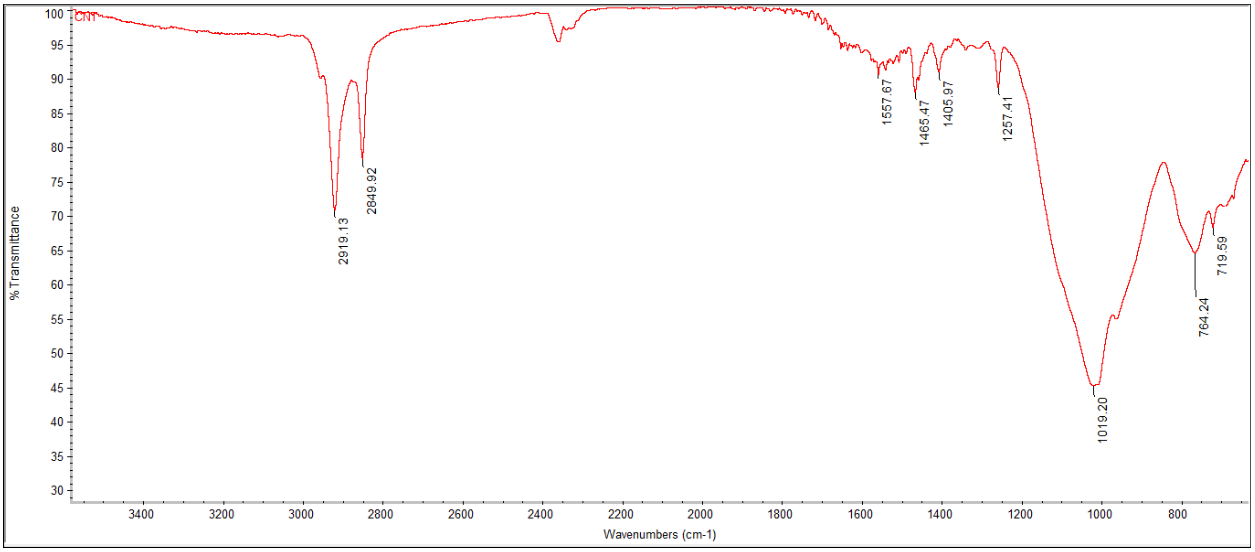

Figure S1-2. FTIR spectrum of the **C-AC** sample.

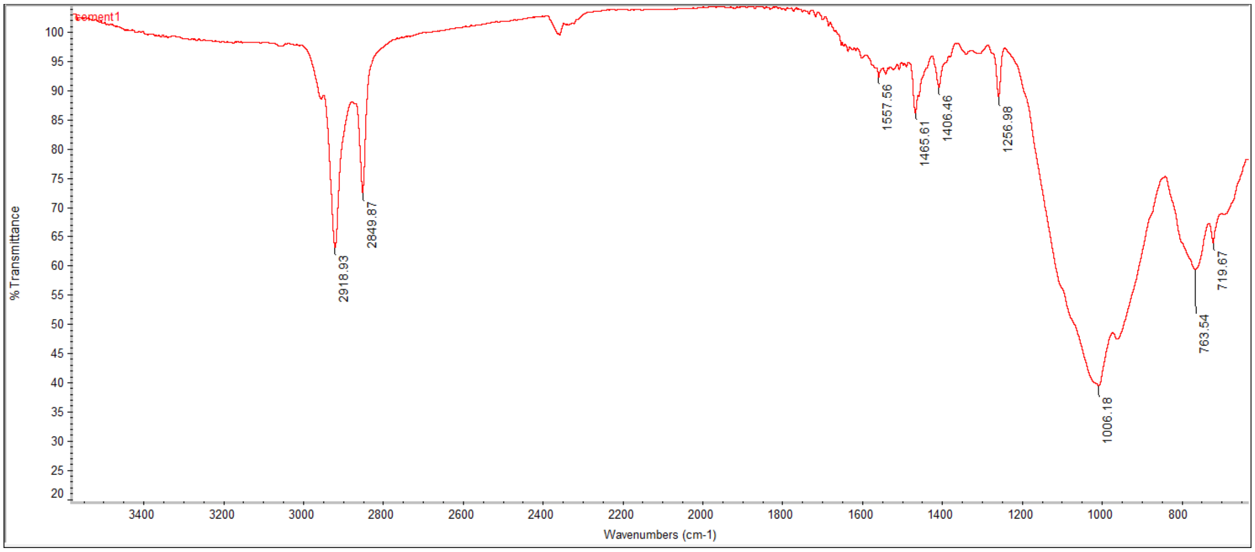

Figure S1-3. FTIR spectrum of the **C-CM** sample.

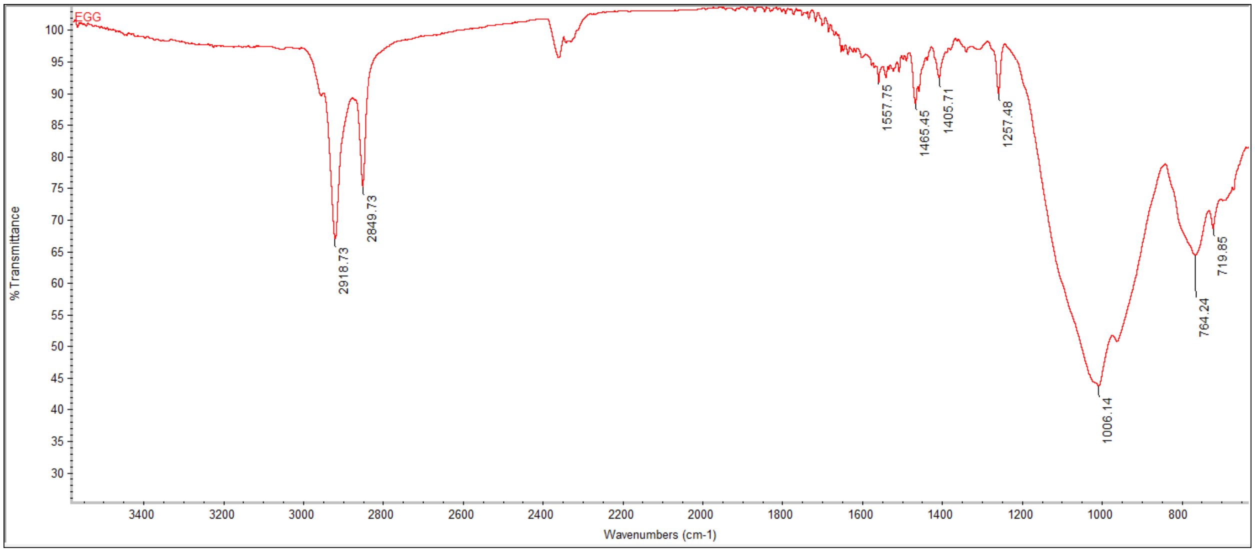

Figure S1-4. FTIR spectrum of the **C-EG** sample.

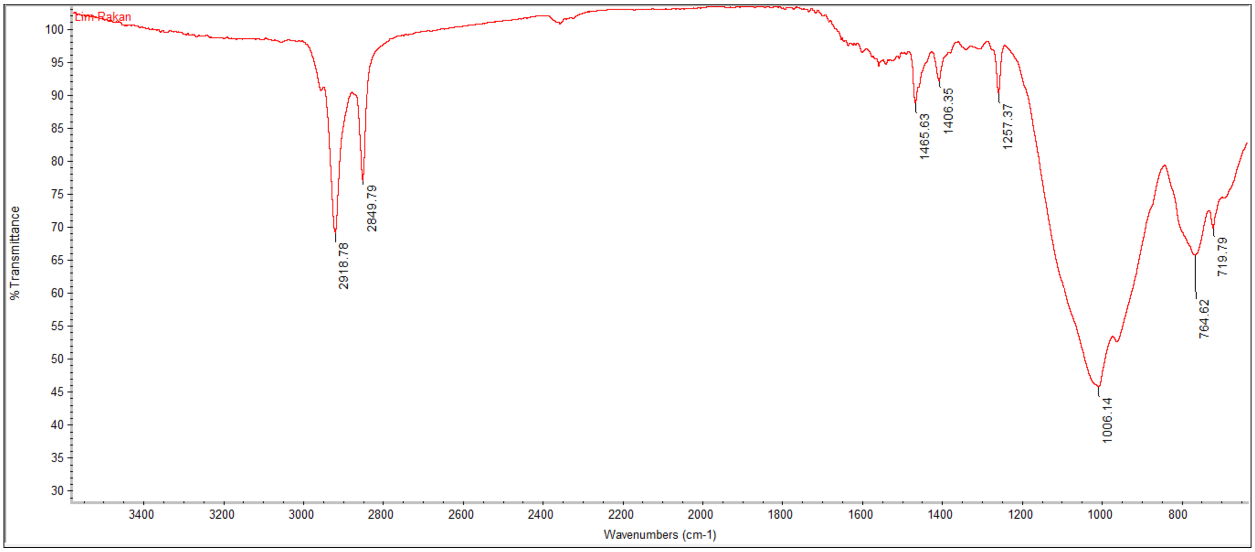

Figure S1-5. FTIR spectrum of the **C-LM** sample.

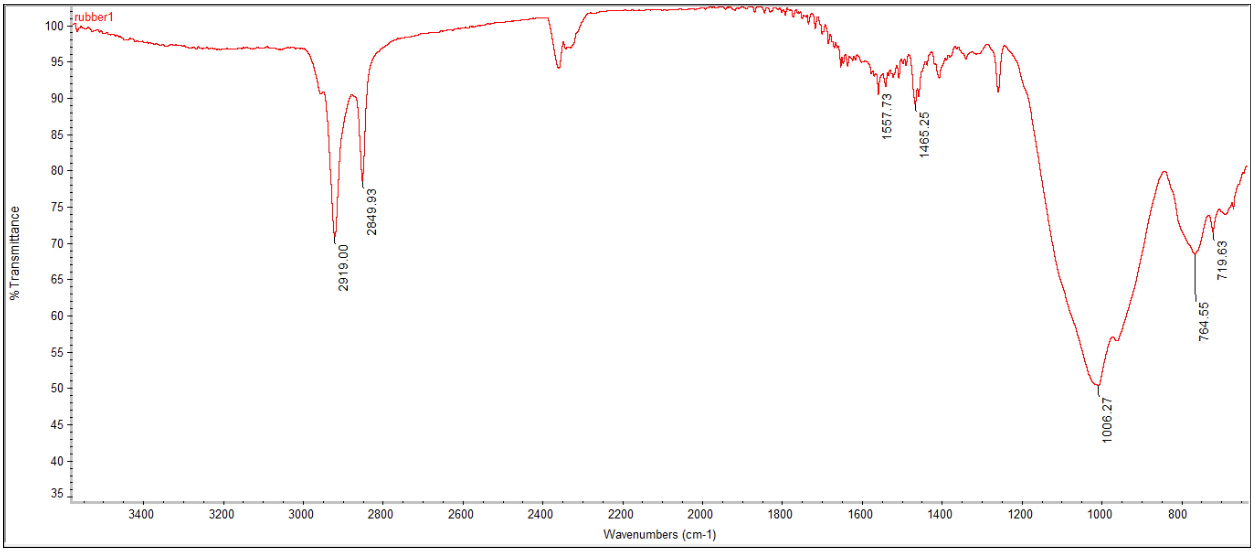

Figure S1-6. FTIR spectrum of the **C-RB** sample.

S2. Representative water contact angle images of all **C** mild steel-coated matrices

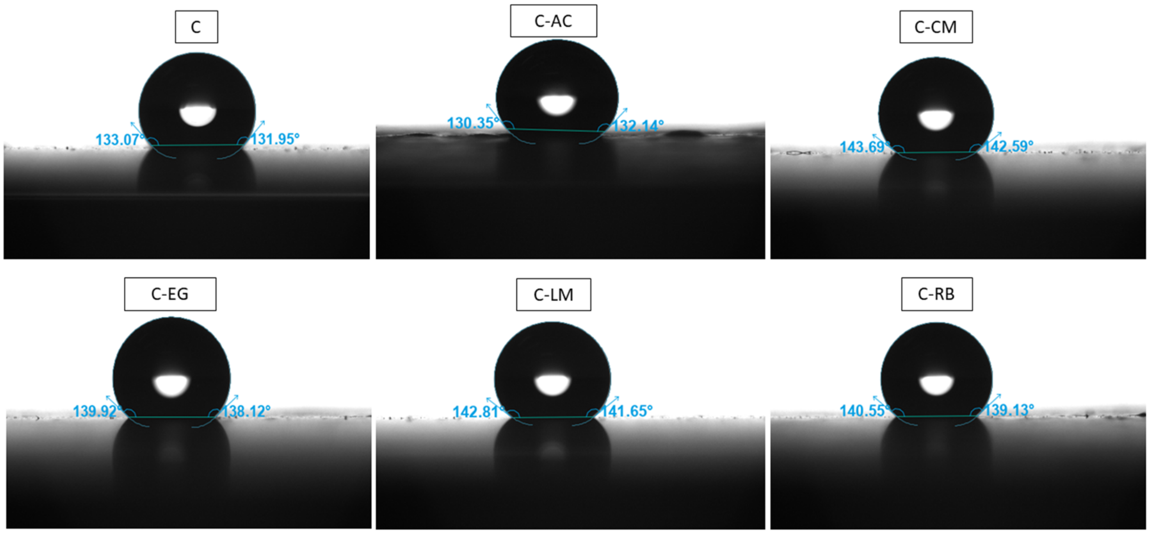

Figure S2-1. Representative contact angle images of the **C** mild steel-coated samples.

S3. Top-surface micrographs of all **C** mild steel-coated matrices immersed in 3.5 wt.% NaCl for 4 weeks and the EDS analysis of the coating layer of the samples

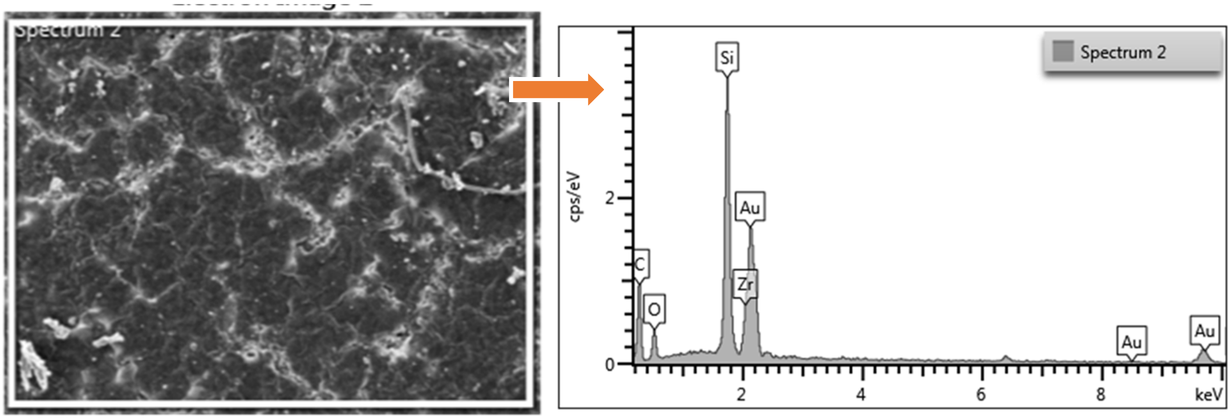

Figure S3-1. Top-surface SEM micrograph of the **C** sample immersed in 3.5 wt.% NaCl for 4 weeks and the EDS analysis of the coating layer of this sample.

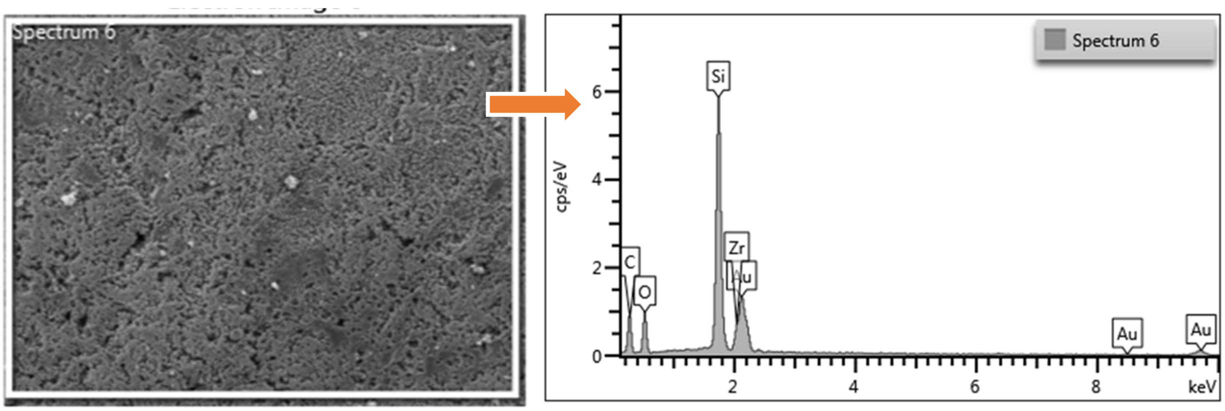

Figure S3-2. Top-surface SEM micrograph of the **C-AC** sample immersed in 3.5 wt.% NaCl for 4 weeks and the EDS analysis of the coating layer of this sample.

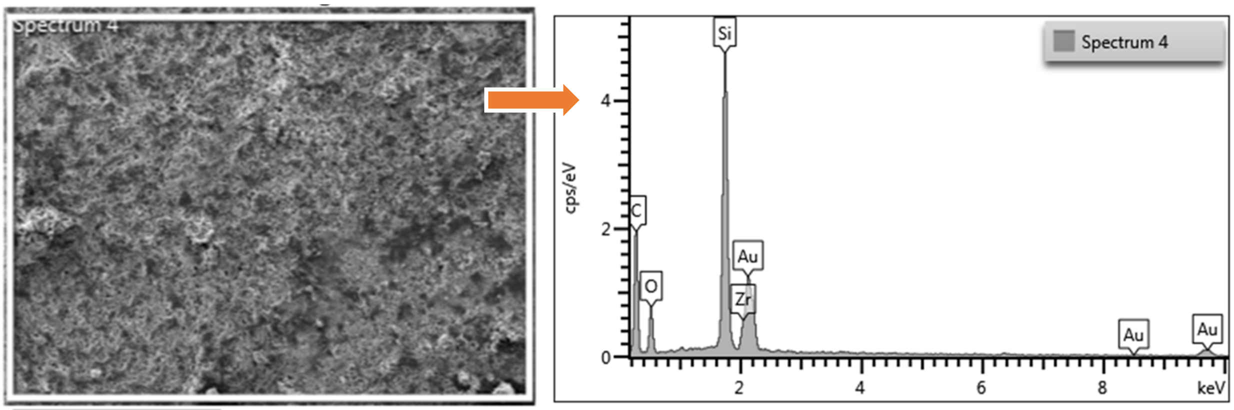

Figure S3-3. Top-surface SEM micrograph of the **C-CM** sample immersed in 3.5 wt.% NaCl for 4 weeks and the EDS analysis of the coating layer of this sample.

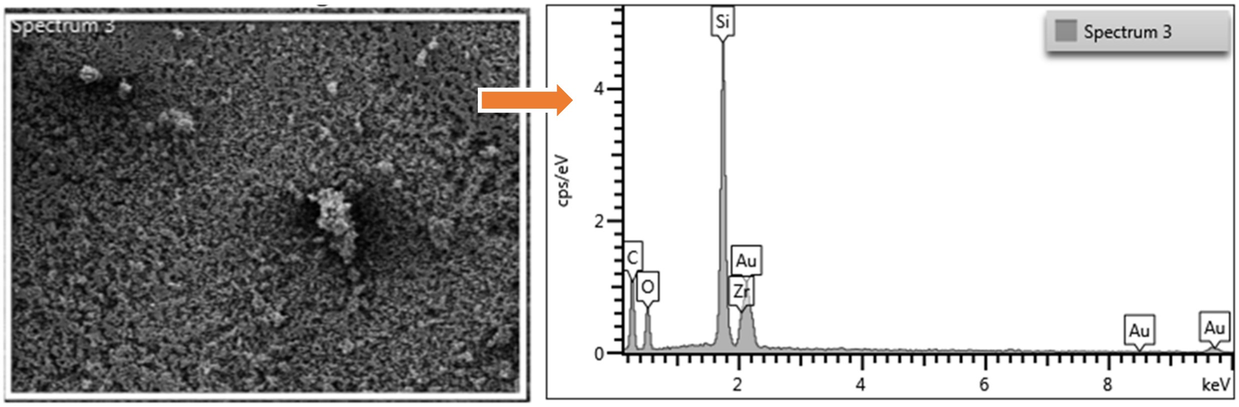

Figure S3-4. Top-surface SEM micrograph of the **C-EG** sample immersed in 3.5 wt.% NaCl for 4 weeks and the EDS analysis of the coating layer of this sample.

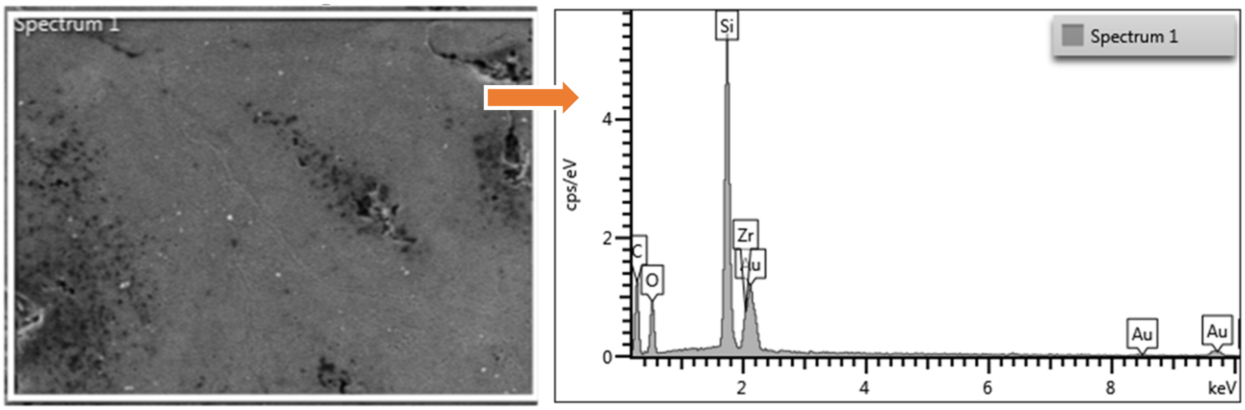

Figure S3-5. Top-surface SEM micrograph of the **C-LM** sample immersed in 3.5 wt.% NaCl for 4 weeks and the EDS analysis of the coating layer of this sample.

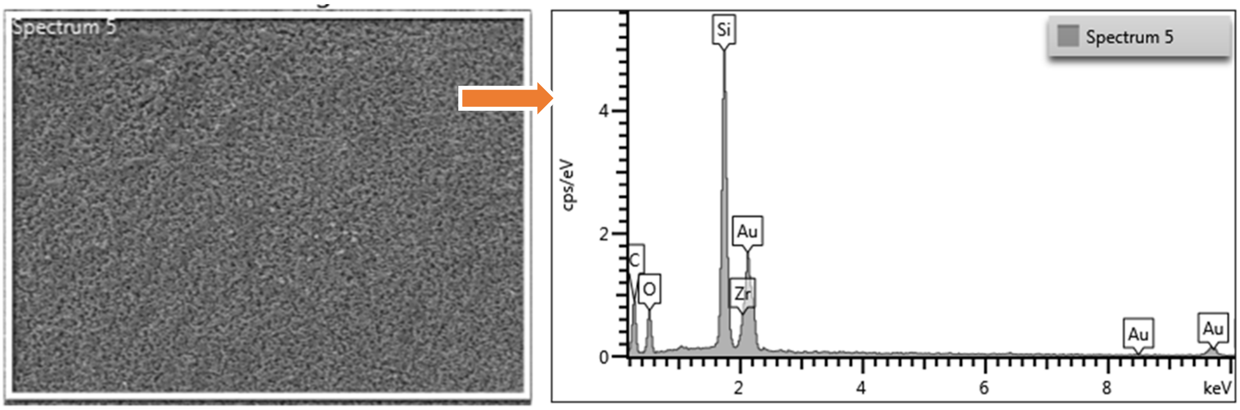

Figure S3-6. Top-surface SEM micrograph of the **C-RB** sample immersed in 3.5 wt.% NaCl for 4 weeks and the EDS analysis of the coating layer of this sample.

S4. Surface roughness typical optical images of all **C** coating matrices on steel using a 3D profilometer

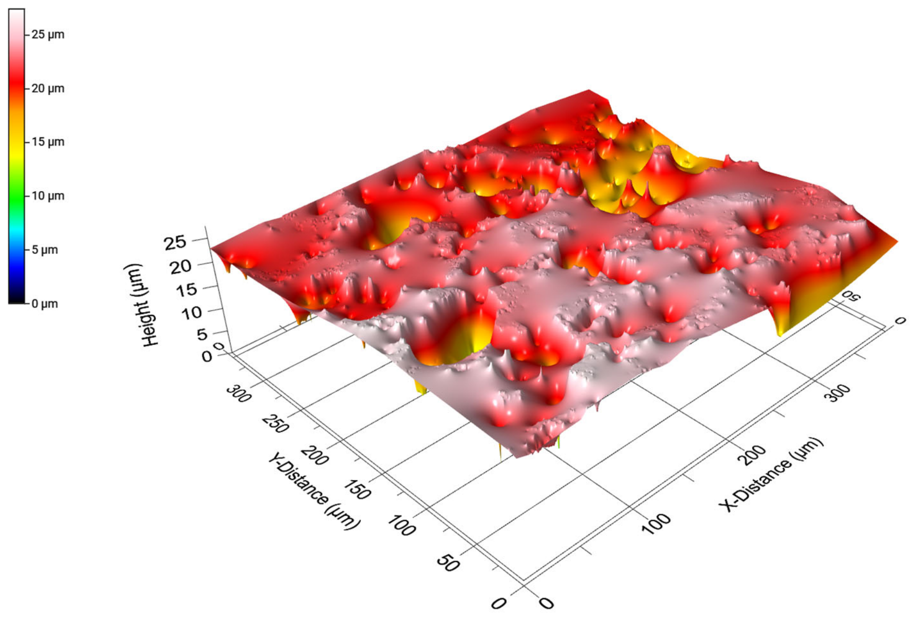

Figure S4-1. Surface roughness optical image of the **C** sample.

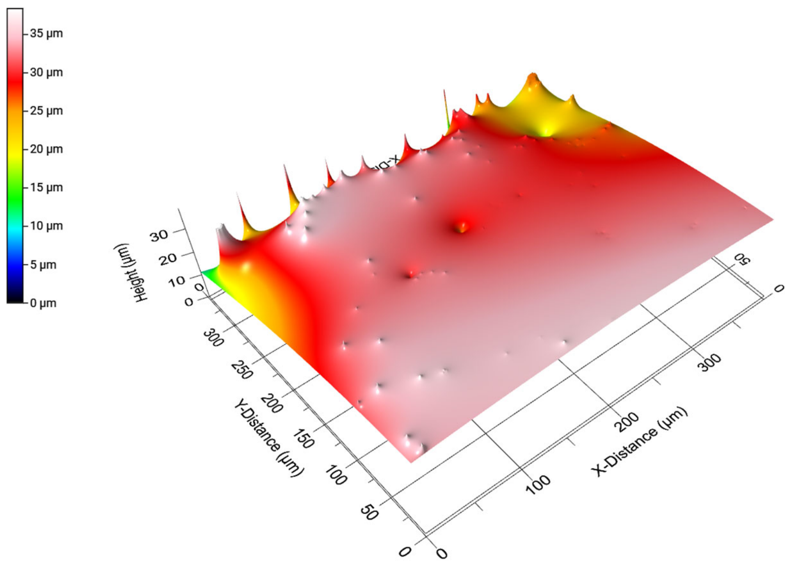

Figure S4-2. Surface roughness optical image of the **C-AC** sample.

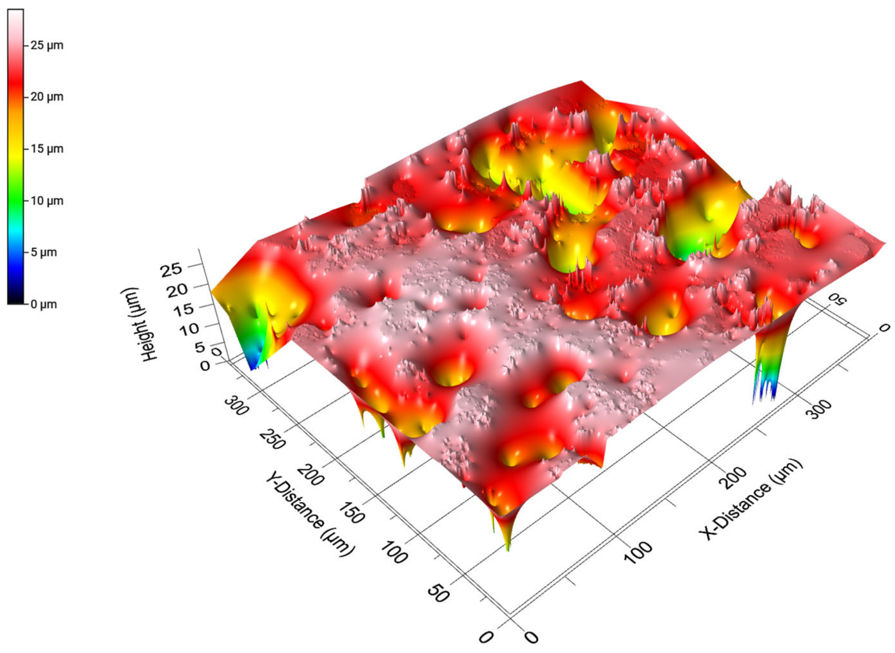

Figure S4-3. Surface roughness optical image of the **C-CM** sample.

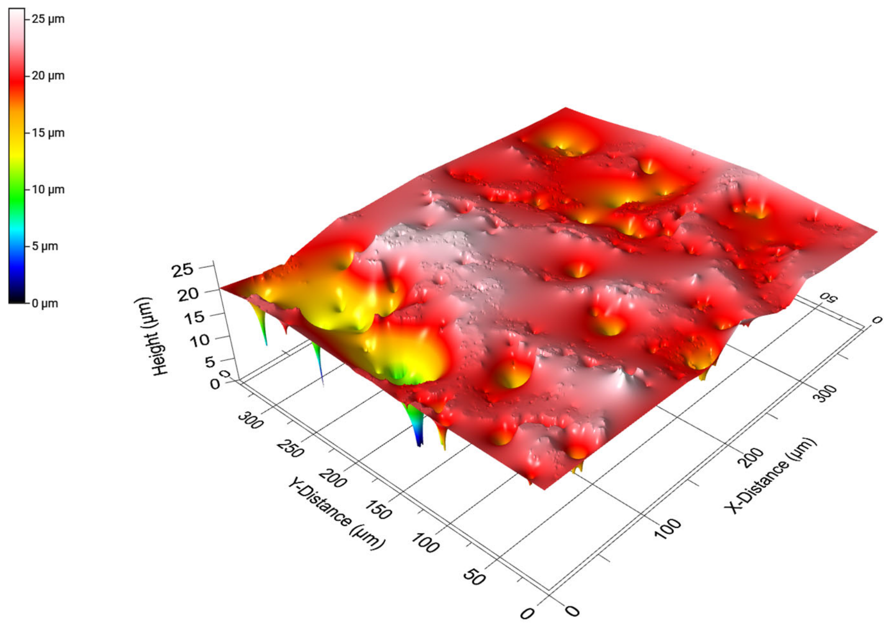

Figure S4-4. Surface roughness optical image of the **C-EG** sample.

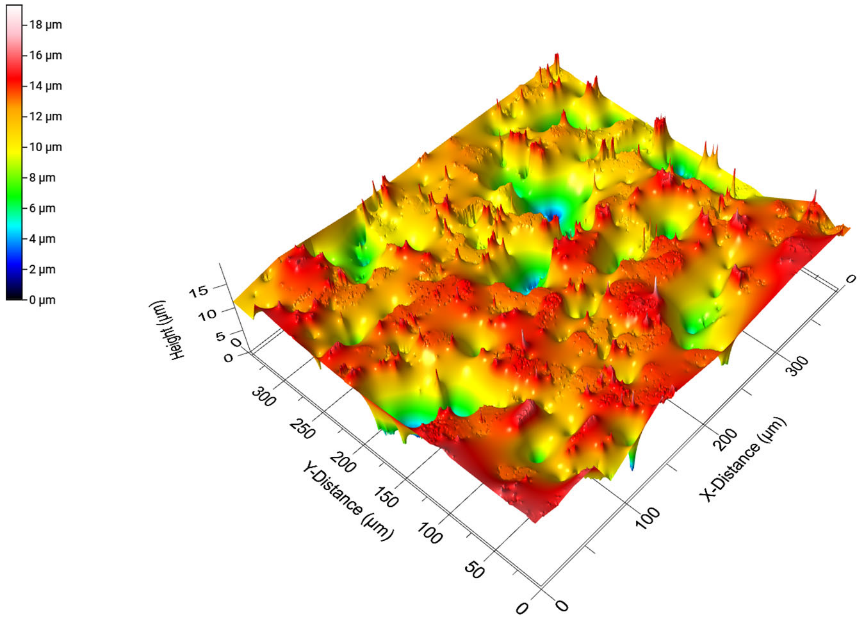

Figure S4-5. Surface roughness optical image of the **C-LM** sample.

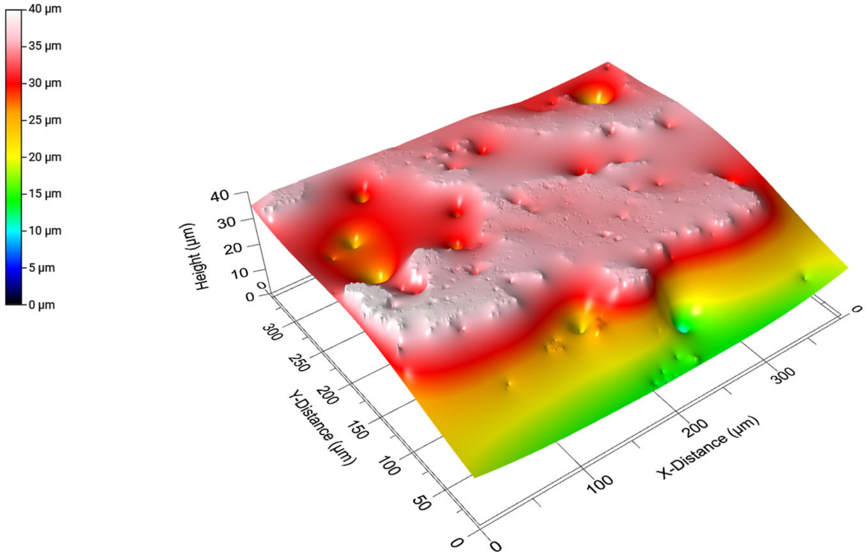

Figure S4-6. Surface roughness optical image of the **C-RB** sample.

S5. Individual Nyquist spectra of all **C** coating matrices after **24 h** of exposure to the 3.5 wt.% NaCl corrosive medium.

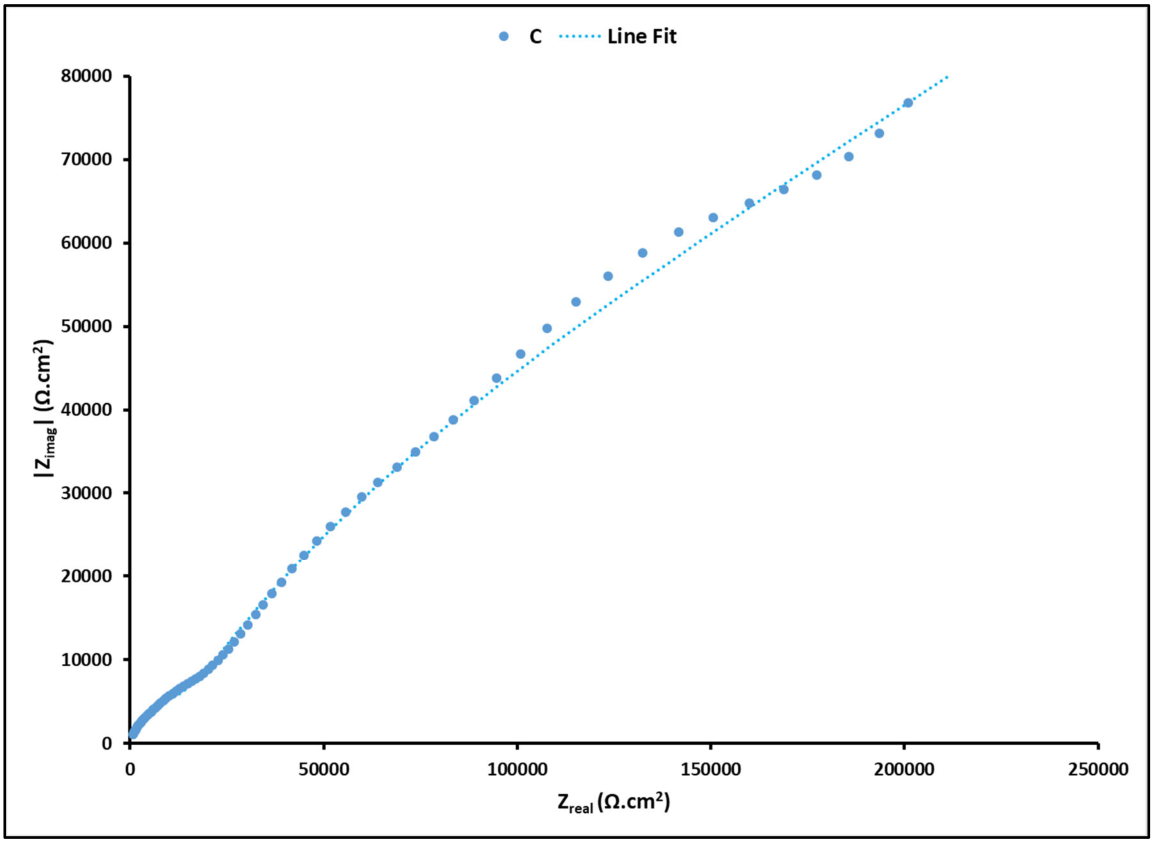

Figure S5-1. EIS Nyquist spectrum of the **C**-coated sample on mild steel substrate after **24 h** of exposure to the 3.5 wt.% NaCl solution.

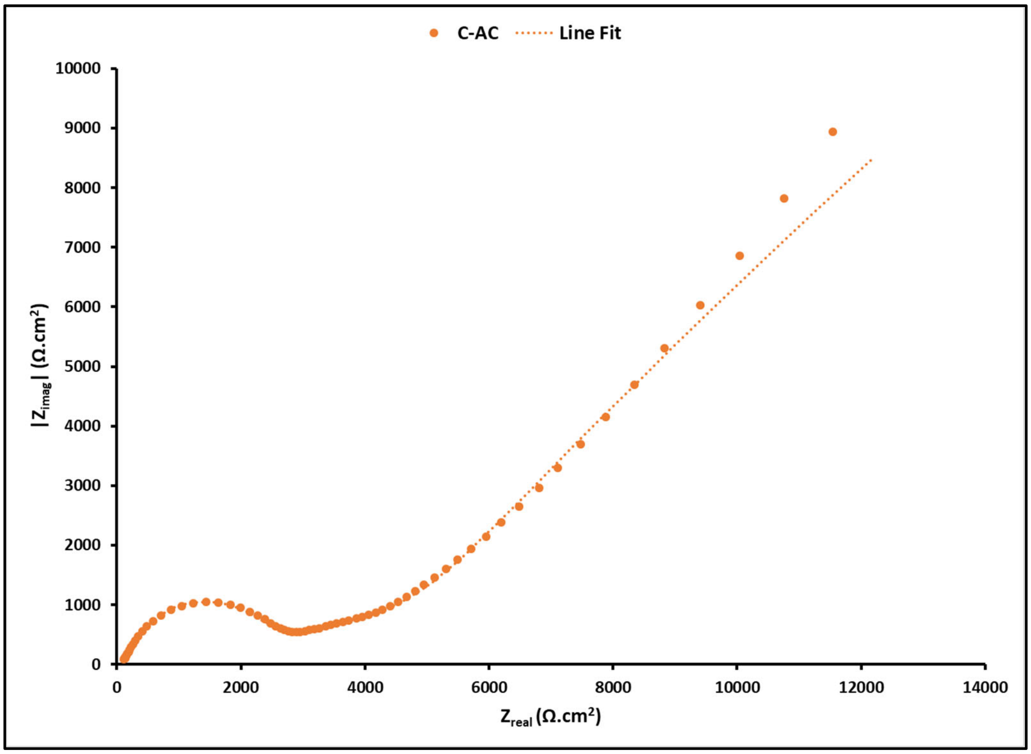

Figure S5-2. EIS Nyquist spectrum of the **C-AC**-coated sample on mild steel substrate after **24 h** of exposure to the 3.5 wt.% NaCl solution.

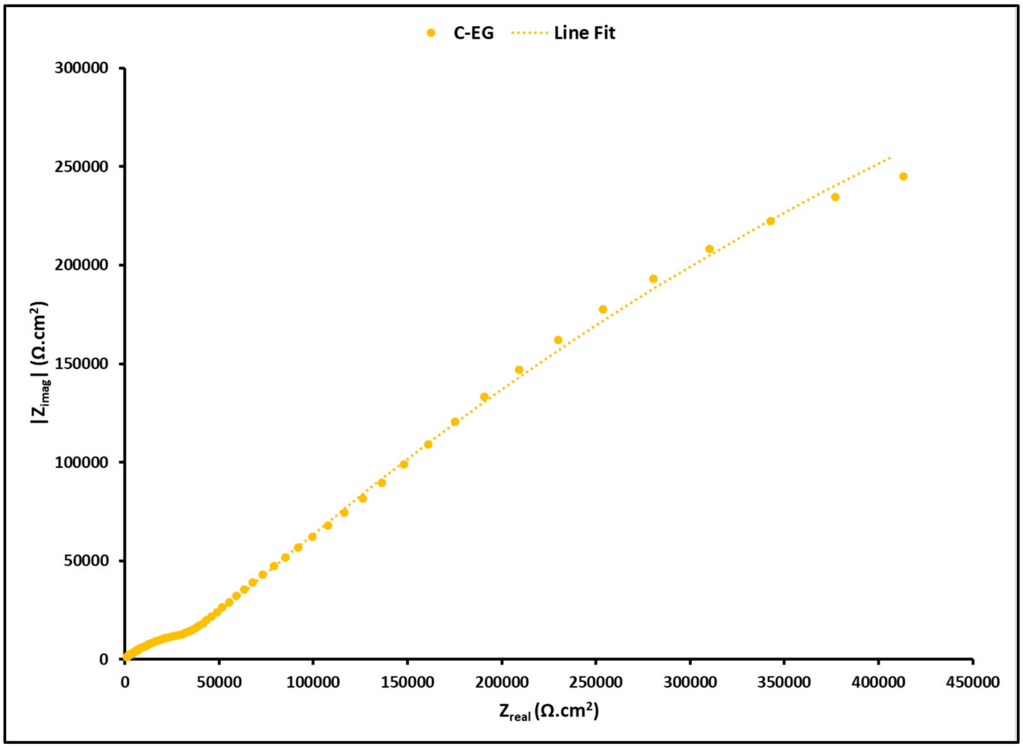

Figure S5-3. EIS Nyquist spectrum of the **C-CM**-coated sample on mild steel substrate after **24 h** of exposure to the 3.5 wt.% NaCl solution.

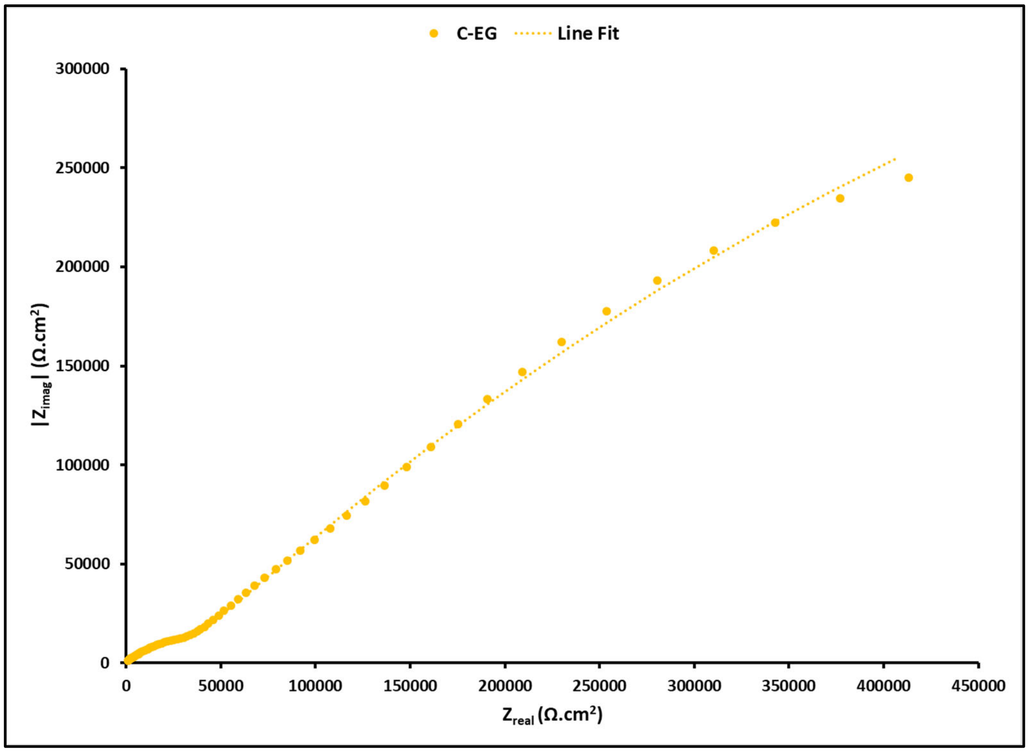

Figure S5-4. EIS Nyquist spectrum of the **C-EG**-coated sample on mild steel substrate after **24 h** of exposure to the 3.5 wt.% NaCl solution.

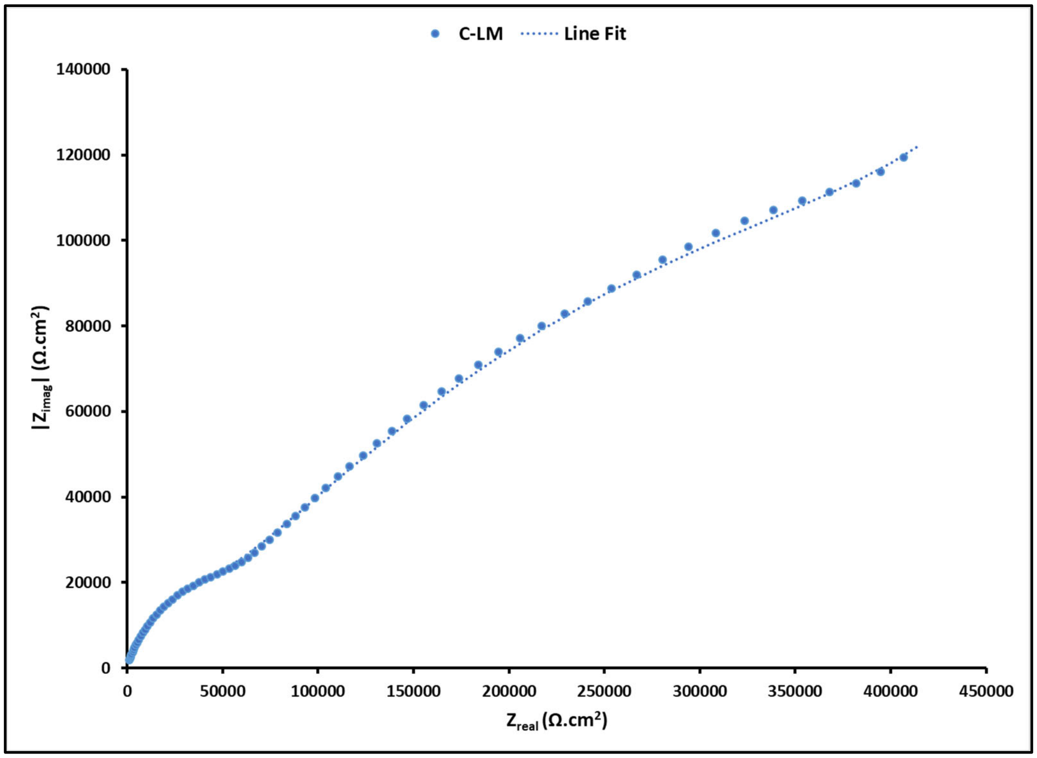

Figure S5-5. EIS Nyquist spectrum of the **C-LM**-coated sample on mild steel substrate after **24 h** of exposure to the 3.5 wt.% NaCl solution.

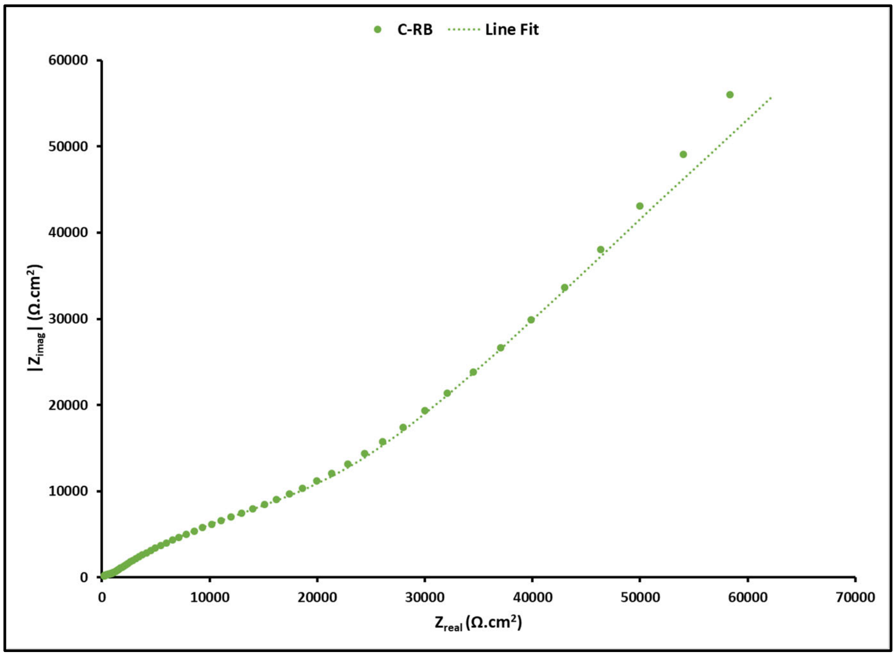

Figure S5-6. EIS Nyquist spectrum of the **C-RB**-coated sample on mild steel substrate after **24 h** of exposure to the 3.5 wt.% NaCl solution.

S6. Individual Nyquist spectra of all **C** coating matrices after **4 weeks** of exposure to the 3.5 wt.% NaCl corrosive medium.

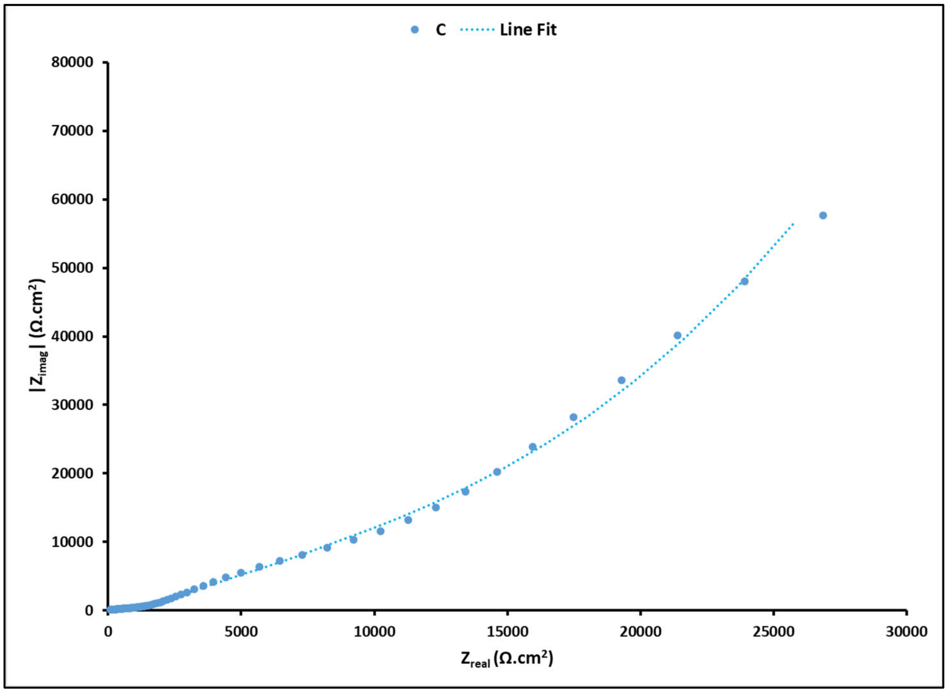

Figure S6-1. EIS Nyquist spectrum of the **C**-coated sample on mild steel substrate after **4 weeks** of exposure to the 3.5 wt.% NaCl solution.

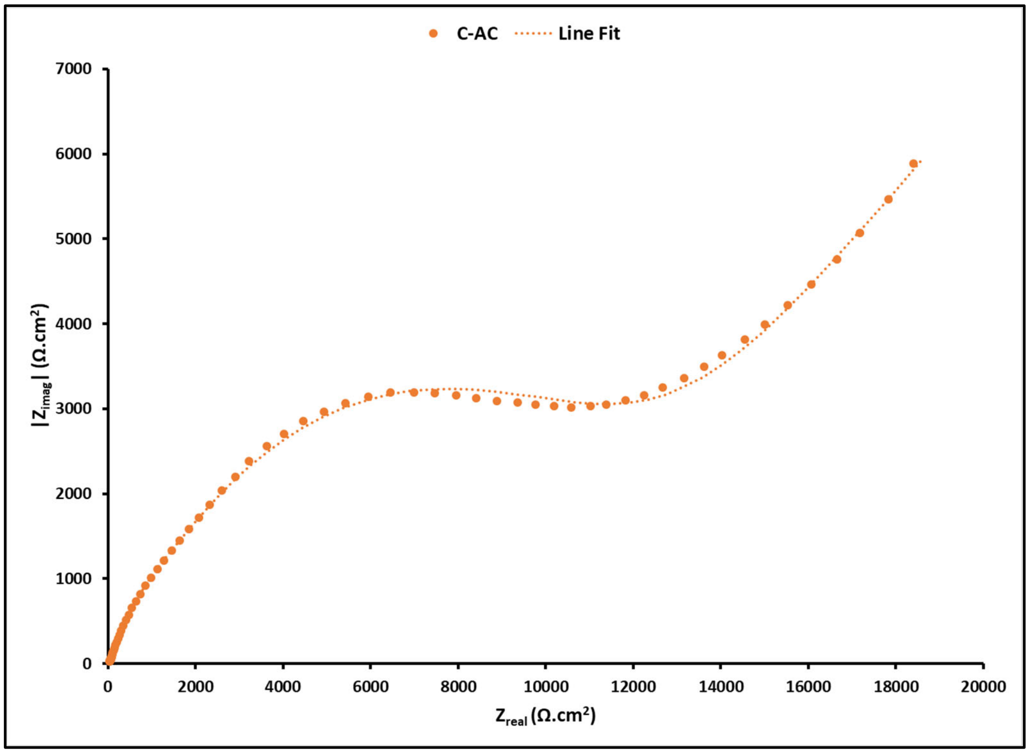

Figure S6-2. EIS Nyquist spectrum of the **C-AC**-coated sample on mild steel substrate after **4 weeks** of exposure to the 3.5 wt.% NaCl solution.

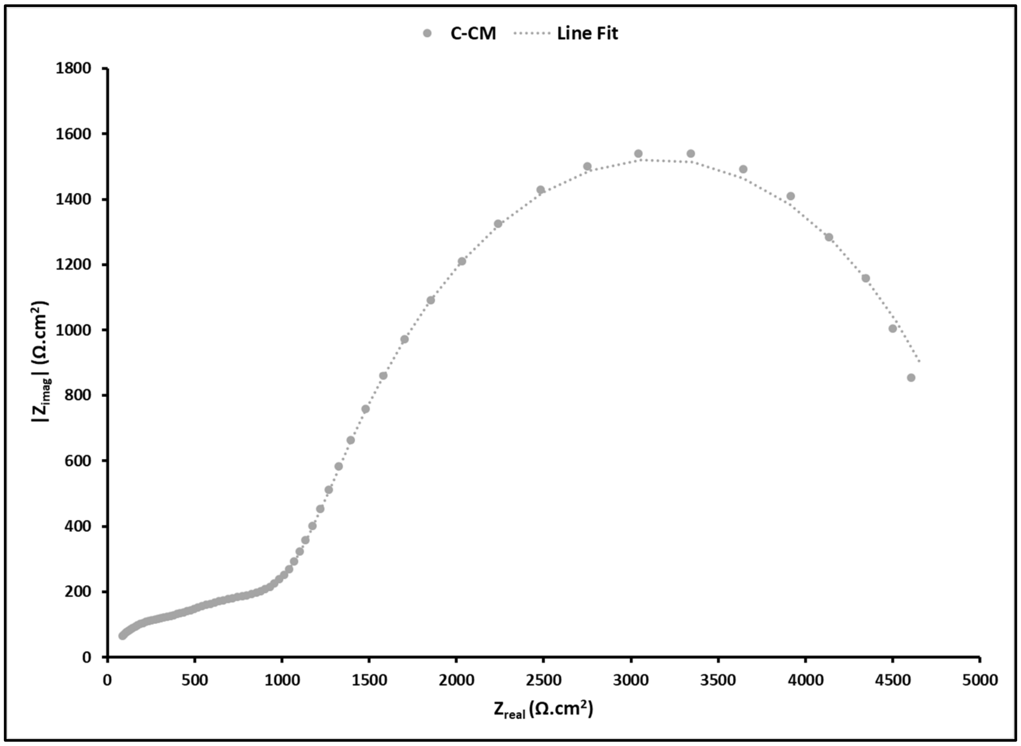

Figure S6-3. EIS Nyquist spectrum of the **C-CM**-coated sample on mild steel substrate after **4 weeks** of exposure to the 3.5 wt.% NaCl solution.

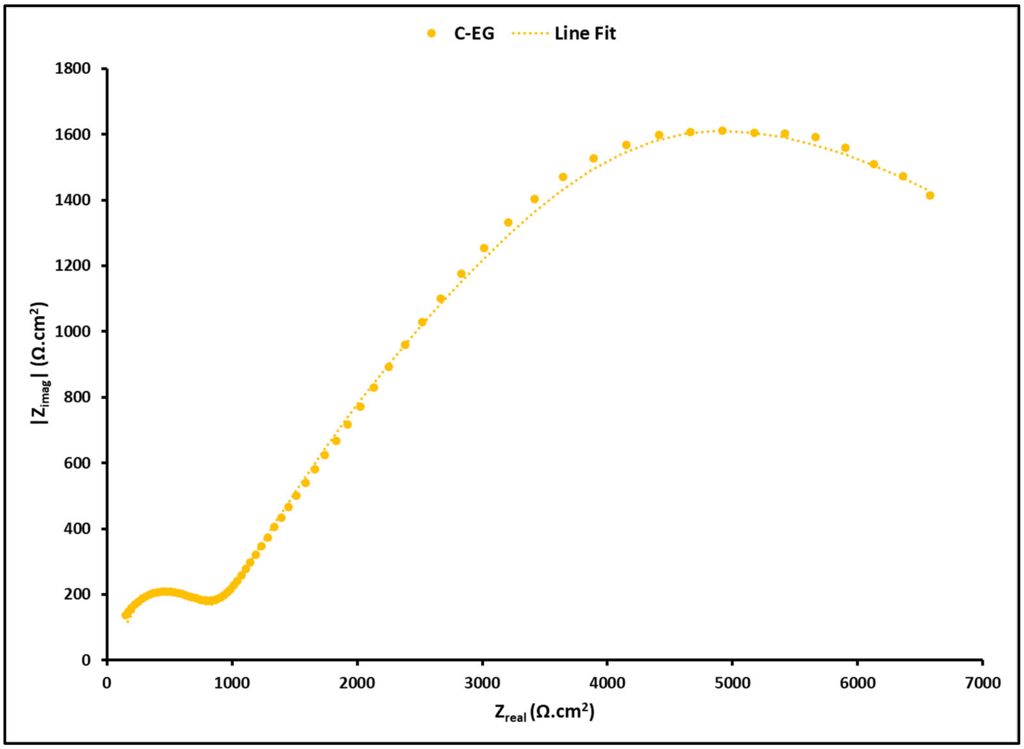

Figure S6-4. EIS Nyquist spectrum of the **C-EG**-coated sample on mild steel substrate after **4 weeks** of exposure to the 3.5 wt.% NaCl solution.

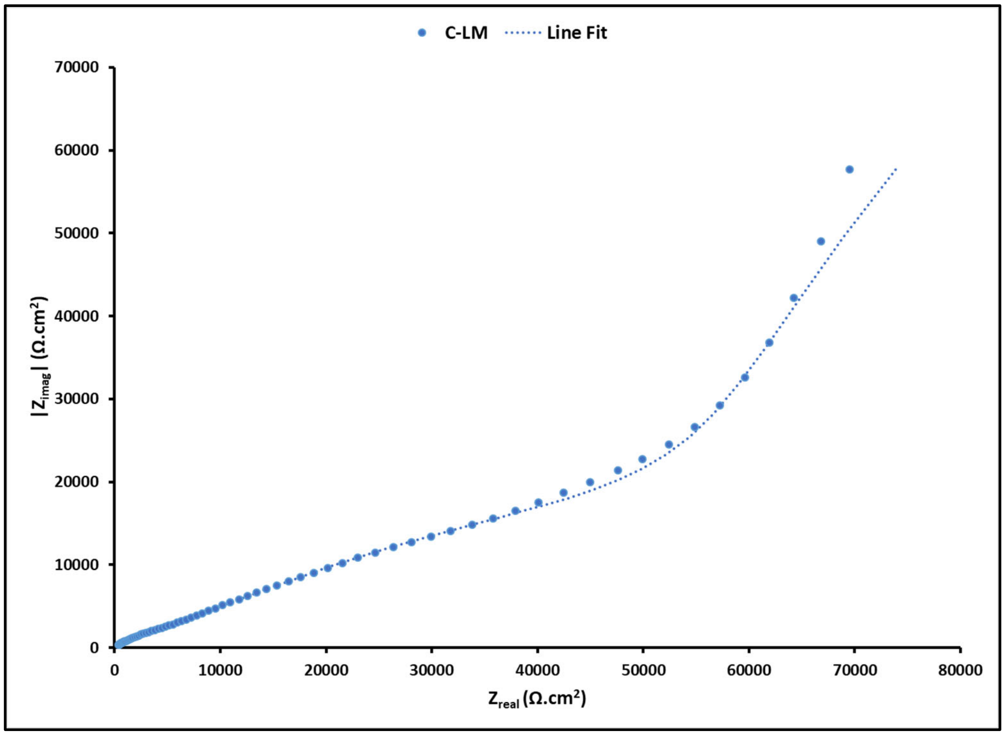

Figure S6-5. EIS Nyquist spectrum of the **C-LM**-coated sample on mild steel substrate after **4 weeks** of exposure to the 3.5 wt.% NaCl solution.

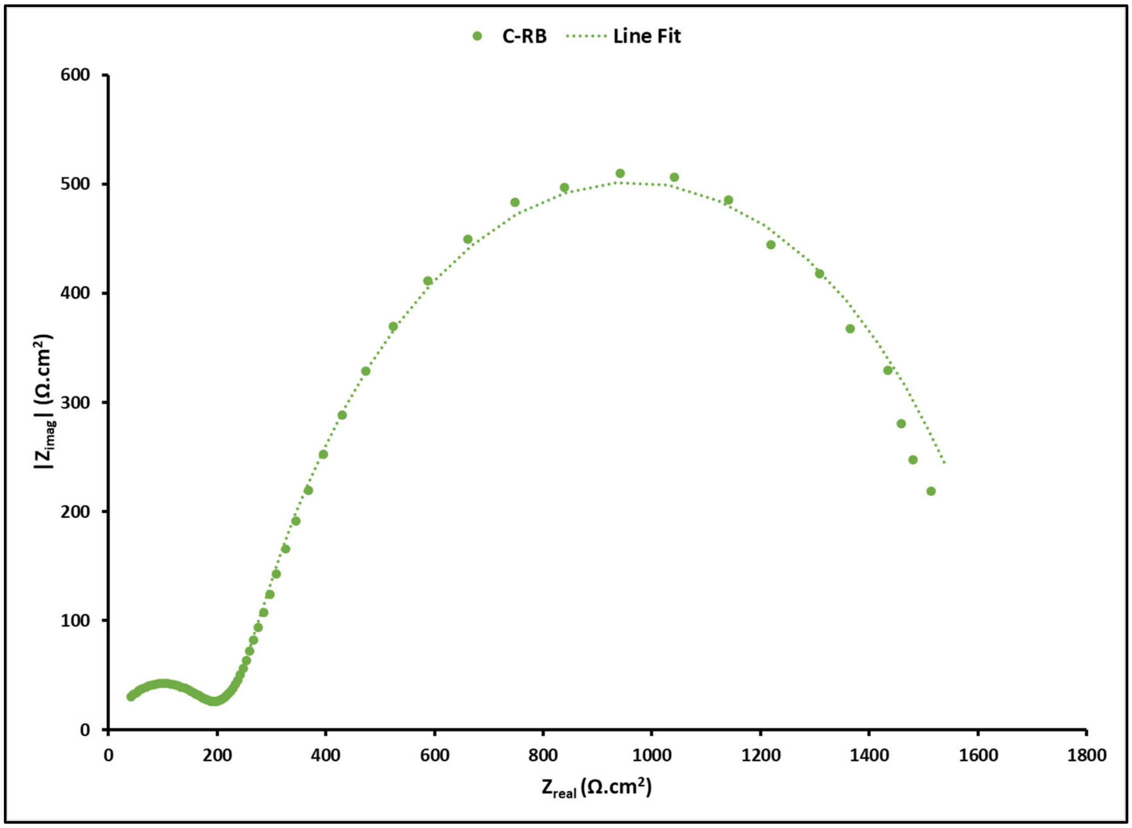

Figure S6-6. EIS Nyquist spectrum of the **C-RB**-coated sample on mild steel substrate after **4 weeks** of exposure to the 3.5 wt.% NaCl solution.

Table S1. Critical load (L_c) for the developed parent and waste-modified mild steel hybrid-coated samples in this study.

**Sample**	**C**	**C-AC**	**C-CM**	**C-EG**	**C-LM**	**C-RB**
**L_c/Failure load (N)**	0.21	0.71	0.48	0.52	0.65	0.50
**Standard deviation**	0.028	0.043	0.046	0.53	0.037	0.053

Table S2. Electrochemical corrosion parameters (corrosion potential, E_{corr}, corrosion current density, i_{corr}) and corrosion rate of mild steel coated with the **C** hybrid sol-gel coatings after *4 weeks* of exposure to a 3.5 wt.% NaCl solution.

Sample	E_{corr} (mV)	I_{corr} ($A.cm^{-2}$)	Corrosion Rate (mpy)
**C**	-131	1.35×10^{-7}	6.160×10^{-3}
**C-AC**	-61	2.37×10^{-6}	0.108
**C-CM**	-423	15.60×10^{-6}	0.712
**C-EG**	-461	7.02×10^{-6}	0.320
**C-LM**	-107	1.42×10^{-7}	0.649
**C-RB**	-500	2.02×10^{-4}	9.216
